# Fosfomycin for Antibiotic Prophylaxis in Men Undergoing a Transrectal Prostate Biopsy: A Systematic Review and Meta-Analysis

**DOI:** 10.3390/medicina59050911

**Published:** 2023-05-10

**Authors:** Hui Mo Gu, Jin Seok Gu, Ho Seok Chung, Seung Il Jung, Dongdeuk Kwon, Myung Ha Kim, Jae Hung Jung, Mi Ah Han, Seung Ji Kang, Eu Chang Hwang, Philipp Dahm

**Affiliations:** 1 Department of Urology, Chonnam National University Medical School, Hwasun 58128, Republic of Korea; heemo1029@hanmail.net (H.M.G.); app9455@naver.com (J.S.G.); hschung615@gmail.com (H.S.C.); drjsi@yahoo.co.kr (S.I.J.); 2 Yonsei Wonju Medical Library, Yonsei University Wonju College of Medicine, Wonju 26426, Republic of Korea; xmankmh@yonsei.ac.kr; 3 Department of Urology, Yonsei University Wonju College of Medicine, Wonju 26426, Republic of Korea; geneuro95@yonsei.ac.kr; 4 Department of Preventive Medicine, College of Medicine, Chosun University, Gwangju 61452, Republic of Korea; mahan@chosun.ac.kr; 5 Department of Infectious Disease, Chonnam National University Medical School, Gwangju 61469, Republic of Korea; sseungi@gmail.com; 6 Department of Urology, University of Minnesota, Minneapolis, MN 55455, USA; pdahm@umn.edu; 7 Minneapolis VA Health Care System, Specialty Care, Minneapolis, MN 55417, USA

**Keywords:** fosfomycin, urinary tract infections, prostate, biopsy

## Abstract

*Background and Objectives:* To assess the effects of fosfomycin compared with other antibiotics as a prophylaxis for urinary tract infections (UTIs) in men undergoing transrectal prostate biopsies. *Materials and Methods:* We searched multiple databases and trial registries without publication language or status restrictions until 4 January 2022. Parallel-group randomized controlled trials (RCTs) and non-randomized studies (NRS) were included. The primary outcomes were febrile UTI, afebrile UTI, and overall UTI. We used GRADE guidance to rate the certainty of evidence of RCTs and NRSs. The protocol was registered with PROSPERO (CRD42022302743). *Results:* We found data on five comparisons; however, this abstract focuses on the primary outcomes of the two most clinically relevant comparisons. Regarding fosfomycin versus fluoroquinolone, five RCTs and four NRSs with a one-month follow-up were included. Based on the RCT evidence, fosfomycin likely resulted in little to no difference in febrile UTIs compared with fluoroquinolone. This difference corresponded to four fewer febrile UTIs per 1000 patients. Fosfomycin likely resulted in little to no difference in afebrile UTIs compared with fluoroquinolone. This difference corresponded to 29 fewer afebrile UTIs per 1000 patients. Fosfomycin likely resulted in little to no difference in overall UTIs compared with fluoroquinolone. This difference corresponded to 35 fewer overall UTIs per 1000 patients. Regarding fosfomycin and fluoroquinolone combined versus fluoroquinolone, two NRSs with a one- to three-month follow-up were included. Based on the NRS evidence, fosfomycin and fluoroquinolone combined may result in little to no difference in febrile UTIs compared with fluoroquinolone. This difference corresponded to 16 fewer febrile UTIs per 1000 patients. *Conclusions:* Compared with fluoroquinolone, fosfomycin or fosfomycin and fluoroquinolone combined may have a similar prophylactic effect on UTIs after a transrectal prostate biopsy. Given the increasing fluoroquinolone resistance and its ease to use, fosfomycin may be a good option for antibiotic prophylaxis.

## 1. Introduction

A definitive diagnosis of prostate cancer depends on histopathological verification of adenocarcinoma in prostate biopsy cores. Therefore, a transrectal ultrasound-guided (TRUS) prostate biopsy has been the most common method for the past 30 years, although it can result in serious infections even in relatively healthy males. In addition, the hospitalization rate for sepsis after a TRUS prostate biopsy is about 3% [1,2]. Thus, prophylaxis for urinary tract infections (UTI) in males undergoing a TRUS prostate biopsy has become paramount.

Various methods have been used to reduce UTIs or infectious complications, including a pre-procedural enema, povidone iodine rectal preparation, rectal swab screening with targeted antibiotic prophylaxis [3,4], switching the antibiotic regimen [5,6,7], and using a transperineal approach instead [1,3].

Traditionally, fluoroquinolones (FQs) were recommended as the first choice for antibiotic prophylaxis. However, UTIs have been rising after prostate biopsies and are thought to be associated with the emergence of FQ-resistant *Escherichia coli* and extended-spectrum β-lactamase producing *E. coli* (ESBL) [1,8,9,10].

A spike in community FQ resistance of 20–30%, especially in Korea [11], has resulted in the need to find an alternative to FQ for prophylactic antibiotics. Fosfomycin is an old antibiotic that has re-emerged as a new strategy to overcome antibiotic resistance without using new drugs. Fosfomycin is an oral bactericidal agent and is a phosphonic acid derivative with a very low molecular weight. It is eliminated mainly unchanged through the kidneys, resulting in very high urinary concentrations within 2–4 h. Therapeutic concentrations in urine are usually maintained for at least 36 h [12]. The high urinary concentrations, combined with fosfomycin’s low molecular weight, may facilitate its diffusion into surrounding tissues such as the prostate gland [12]. While there is limited data explicitly demonstrating fosfomycin’s concentration in prostate tissue [13], the drug has proven effective in treating prostatitis [14].

Recent randomized controlled trials (RCTs) and non-randomized observational studies (NRSs) have shown the superior prophylactic effect of fosfomycin on UTIs after TRUS prostate biopsies compared with FQ or other antibiotics [6,15,16,17]. Moreover, some systematic reviews (SRs) have demonstrated the equivalence or superiority of fosfomycin in lowering infectious complications after a TRUS prostate biopsy compared with FQ and other antibiotics [7,18,19,20]. However, these SRs have not evaluated the risk of bias in the individual studies nor rated the certainty of the evidence using the GRADE approach [21]. Furthermore, since 2019, several prospective and retrospective observational studies have been published; therefore, an additional summary of the evidence for using fosfomycin as antibiotic prophylaxis after a TRUS prostate biopsy is needed. Therefore, to provide the current body of evidence and assist medical professionals, this study aimed to investigate the prophylactic effects and adverse drug events of fosfomycin versus other antibiotics on UTIs after a TRUS prostate biopsy.

## 2. Materials and Methods

### 2.1. Protocol Registration and Eligibility Criteria

This systematic review and meta-analysis was based on a priori registered protocol (PROSPERO: CRD42022302743). This study was performed according to the Preferred Reporting Items for Systematic Reviews and Meta-Analyses (PRISMA) guidelines and AMSTAR 2 Checklist.

The following inclusion criteria were used to identify relevant studies:

- Type of studies: RCTs, which provide a higher certainty of the evidence, and NRSs, which are similar to the relevant RCTs, as a source of complementary, sequential, or replacement evidence for the RCTs, were included regardless of their status, date, or language of publication.
- Types of participants: Studies on males undergoing TRUS prostate biopsies who were suspected of prostate cancer by a prostate-specific antigen (PSA) and digital rectal exam (DRE) were included.
- Types of interventions: Studies that used fosfomycin as a prophylactic antibiotic (versus FQ or other types of antibiotics) to prevent UTIs or infectious complications after TRUS prostate biopsies were considered, regardless of the use of other adjunctive therapies (i.e., povidone rectal cleansings or rectal enemas), provided these were consistent in both groups.

### 2.2. Outcomes

The measurement of the outcomes assessed in this review was not used as an inclusion criterion. Primary outcomes were febrile UTI, afebrile UTI, and overall UTI. Secondary outcomes were adverse drug events, positive urine and blood cultures, and FQ resistance. All outcomes were measured within 30 days after the prostate biopsy. The clinically important differences were used to rate the overall certainty of the evidence in the ‘Summary of findings’ table [22]. There is no reported threshold for the review outcomes. The clinically important differences for febrile UTI, positive urine culture, positive blood culture, and fluoroquinolone resistance were considered as an absolute risk reduction or increase of 2%. The clinically important differences for afebrile UTI, overall UTI, and adverse drug events were used as an absolute risk reduction or increase of 5%. These risk reductions and increases were decided on by the input of the clinical expertise of the infectious disease internal medicine doctor on the review team.

### 2.3. Search Method for Identification and Selection of the Studies

A comprehensive literature search was performed up to 14 January 2022, using a range of established scientific databases (MEDLINE [Ovid], Cochrane, Embase, Web of Science, Koreamed, and Kmbase) and trial registries (ClinicalTrials.gov and World Health Organization International Clinical Trials Registry Platform) regardless of their publication status or the language of publication. The references of the full articles retrieved for our review were also searched to identify any additional studies. All steps were performed independently and in duplicate following the protocol using the Covidence software platform (www.covidence.org, accessed on 16 January 2022). Details of the search strategies are in Supplementary Materials. Two review authors (HMG and ECH) independently investigated all the potentially relevant records as full-text mapped records to studies and classified studies as included studies, excluded studies, studies awaiting classification, or ongoing studies, following the criteria for each provided in the Cochrane Handbook [23]. Disagreements between the reviewers, if not resolved by discussion, were determined by consultation with a third author (JHJ). A PRISMA flow diagram showing the study selection process is presented [24].

### 2.4. Data Extraction and Risk of Bias Assessment

For studies that fulfilled the inclusion criteria, three review authors (HMG, JSG, and ECH) independently extracted information on study design, study dates, setting, country, participant characteristics, intervention details, comparisons, outcomes, funding sources, and conflict of interest. No additional information was required beyond the published data. The risk of bias for RCTs was assessed using a recently developed version of the Cochrane ‘Risk of bias’ tool (RoB 2) [25]. Modified Newcastle-Ottawa scale criteria were used to assess the quality of the NRSs [26]. Two review authors (HMG and ECH) independently evaluated the risk of bias and study quality, considering the effect of the assignment on the intervention. When the two authors disagreed, a final consensus was decided on by the rating of a third author (JHJ).

### 2.5. Data Synthesis and Analysis

The data were summarized using a random-effects model. The Review Manager 5.4.1 software (Cochrane Collaboration, Copenhagen, Denmark) was used for the statistical analyses. Since the outcomes were all dichotomous, the risk ratio (RR) with a 95% confidence interval was calculated. Heterogeneity (inconsistency) was identified by visually inspecting the forest plots to assess the overlap of CIs and the I^2^ statistic. The I^2^ statistic was interpreted following the guidance of the Cochrane Handbook [23]. When heterogeneity was found, an attempt to determine its possible causes was made by examining the subgroup analysis. However, a subgroup analysis could not be performed due to the insufficient available data from the included studies. Additionally, the small study effects could not be assessed using a funnel plot since there were less than ten included studies in each comparison [23]. A sensitivity analysis was attempted as per the protocol; however, no sensitivity analysis was possible since the included studies were mostly unclear and had a high risk of bias.

### 2.6. Summary of Findings Table

The certainty of evidence (CoE) was rated on a per-outcome basis using the Grading of Recommendations Assessment, Development, and Evaluation (GRADE) framework, which considers five criteria related to the internal (risk of bias, inconsistency, imprecision, and publication bias) and external (directness of results) validities [21]. For each comparison, two review authors (ECH, JHJ) independently rated the certainty of the evidence for each outcome as ‘high,’ ‘moderate,’ ‘low,’ or ‘very low’ using the GRADEpro software [27] and constructed a summary of findings table. Discrepancies were resolved by consensus. For each comparison, these tables provided key information about the best estimate of relative and absolute effects for each outcome [28]. The GRADE guidance was used to describe the certainty of the evidence and the magnitude of the effect size [29].

### 2.7. Ethics Statement

Ethical approval was waived due to the nature of this study.

## 3. Results

### 3.1. Search Results

**Figure 1:**
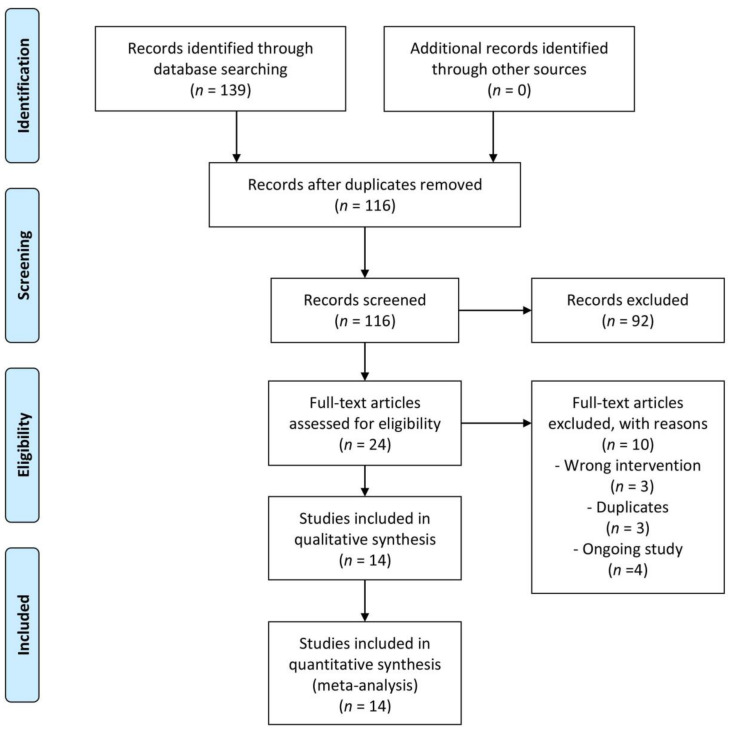
Preferred Reporting Items for Systematic Reviews and Meta-Analyses (PRISMA) flow chart.

The search yielded 139 studies. After removing duplicates, the titles and abstracts of 116 studies were screened. After excluding 92 studies (the 92 studies’ titles and abstracts were irrelevant to the review question; men undergoing a transrectal prostate biopsy (population); fosfomycin (intervention); other antibiotics (comparison)), 24 full-text articles were screened, of which ten studies were excluded that did not meet the inclusion criteria. In total, 14 studies (six RCTs [15,16,30,31,32,33] and eight NRSs, including abstracts [6,17,34,35,36,37,38,39]) were included in the qualitative and quantitative synthesis of this review. See the PRISMA flowchart (Figure 1).

### 3.2. Description of the Included Studies

For details, please refer to Supplementary Table S1 of the characteristics of the included studies. In total, 2636 randomized and 6352 non-randomized patients suspected of prostate cancer by PSA (mostly above 4 ng/mL) and DRE were included in this study. The age range of the participants was 58 to 76 years, and most of the studies measured the outcomes 30 days after the TRUS biopsy. Two studies used periprostatic nerve block using lidocaine or bupivacaine [17,33]. In addition, participants from eight studies received an enema before or on the day of the TRUS biopsy [6,30,31,34,35,37,38,39].

Five RCTs [16,30,31,32,33] and four NRSs [6,17,34,35] compared fosfomycin with FQ. Most studies used a single dose of fosfomycin before the TRUS biopsy [16,17,31,32,33,34,35], but two studies used two doses of fosfomycin (before and after the TRUS biopsy) [6,30]. However, the administration dose and duration of FQ differed between studies.

Two NRSs compared fosfomycin combined with FQ and FQ [38,39]. The fosfomycin dose was the same between the studies, but the FQ regimen differed. In one study, the FQ was given intravenously [39] while in the other, it was given orally [38]. Two NRSs and one RCT compared fosfomycin with different antibiotics, namely β-lactam or FQ [37], FQ and metronidazole [15], or FQ, metronidazole, and gentamycin [36].

Seven studies specified the funding source [15,17,34,35,37,38,39], and 11 reported conflicts of interest [6,15,16,17,30,31,33,34,37,38,39].

### 3.3. Risk of Bias and Quality of the Included Studies

**Figure 2:**
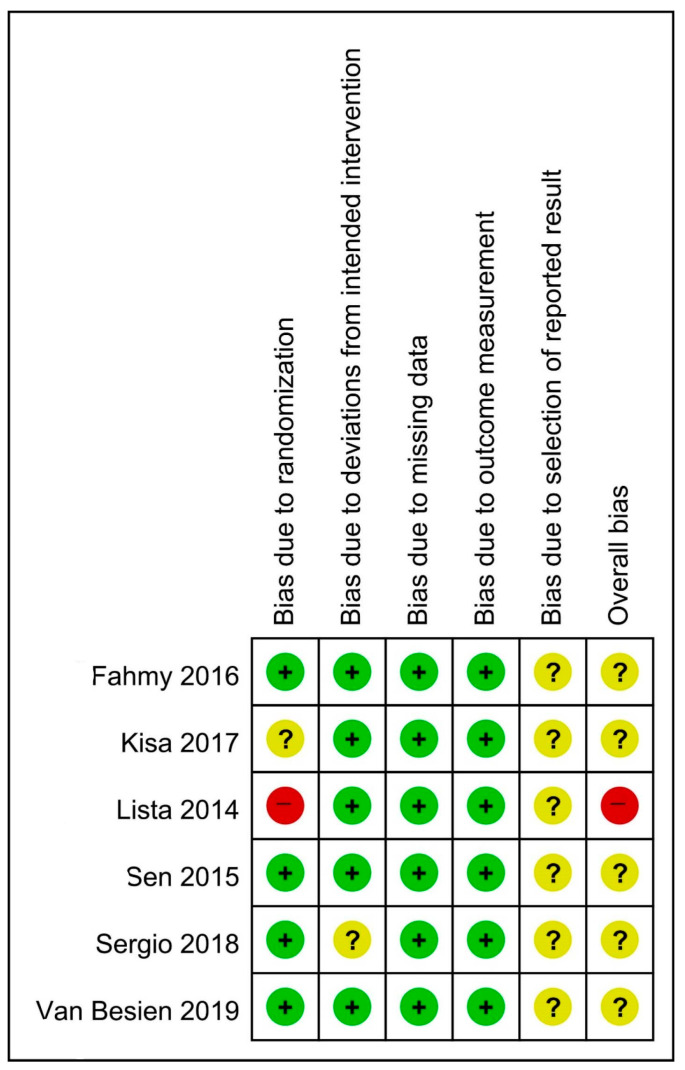
Risk of bias summary for RCTs, review authors’ judgments about each risk of bias item for each included study; Green: low risk of bias; Yellow: some concern; Red: high risk of bias [15,16,30,31,32,33].

Five RCTs were found to have some concerns of overall bias due to randomization [16], deviation from the intended intervention [32], and selection of the reported result [15,16,31,32,33]. One RCT had a high risk of overall bias rating due to the high risk of randomization and some concerns about reporting bias [30]. The included NRSs were moderate [36] to good quality [6,17,34,35,37,38,39]. Since the study by Yang et al. was only an abstract, there was uncertainty about the identification of the exposure and assessment of the outcome [36]. The risk of bias summary and quality assessment of the included studies is summarized in Figure 2 and Appendix A, Table A1.

### 3.4. Main Analysis Based on the Two Most Clinically Relevant Comparisons

#### 3.4.1. Fosfomycin versus FQ

Please refer to Table 1, Appendix A, Table A2, and Supplementary Figures S1–S7. The summary of findings table for NRSs is not presented since the certainty of the evidence was lower than the RCTs.

##### Primary Outcomes

(1) Febrile UTI

Five RCTs with 1511 patients (fosfomycin *n* = 784, FQ *n* = 727) reported febrile UTIs [16,30,31,32,33]. There is probably little to no difference in febrile UTI incidence between fosfomycin and FQ (RR 0.84, 95% CI 0.42 to 1.69; I^2^ = 0%; moderate-certainty evidence). We downgraded the certainty of the evidence due to serious study limitations (−1).

Four NRSs with 2513 patients (fosfomycin *n* = 1231, FQ *n* = 1282) reported febrile UTIs [6,17,34,35]. We are very uncertain whether fosfomycin results in more or fewer febrile UTIs than FQ (RR 0.37, 95% CI 0.11 to 1.24; I^2^ = 73%; very low-certainty evidence). The certainty of the evidence was downgraded for inconsistency (−1) and imprecision (−1).

Based on the evidence from the RCTs that provided evidence of higher certainty, there is probably little to no difference in febrile UTI incidence between fosfomycin and FQ (moderate-certainty evidence).

(2) Afebrile UTI

Five RCTs with 1511 patients (fosfomycin *n* = 784, FQ *n* = 727) reported afebrile UTIs [16,30,31,32,33]. There is probably little to no difference in afebrile UTI incidence between fosfomycin and FQ (RR 0.43, 95% CI 0.23 to 0.78; I^2^ = 0%; moderate-certainty evidence). We downgraded the certainty of the evidence for serious study limitations (−1).

Three NRSs with 1926 patients (fosfomycin *n* = 817, FQ *n* = 1109) reported afebrile UTIs [6,17,34]. We are very uncertain whether fosfomycin results in fewer afebrile UTIs than FQ (RR 0.30, 95% CI 0.14 to 0.66; I^2^ = 38%; very low-certainty evidence). We downgraded the certainty of the evidence for imprecision (−1).

Based on the evidence from the RCTs that provided evidence of higher certainty, there is probably little to no difference in afebrile UTI incidence between fosfomycin and FQ (moderate-certainty evidence).

(3) Overall UTI

Five RCTs with 1511 patients (fosfomycin *n* = 784, FQ *n* = 727) reported overall UTIs [16,30,31,32,33]. There is probably little to no difference in overall UTI incidence between fosfomycin and FQ (RR 0.53, 95% CI 0.31 to 0.92; I^2^ = 25%; moderate-certainty evidence). We downgraded the certainty of the evidence for serious study limitations (−1).

Three NRSs with 1926 patients (fosfomycin *n* = 817, FQ *n* = 1109) reported overall UTIs [6,17,34]. We are very uncertain whether fosfomycin results in fewer overall UTIs than FQ (RR 0.29, 95% CI 0.11 to 0.80; I^2^ = 79%; very low-certainty evidence). We downgraded the certainty of the evidence for inconsistency (−1) and imprecision (−1).

Based on the evidence from the RCTs that provided evidence of higher certainty, there is probably little to no difference in overall UTI incidence between fosfomycin and FQ (moderate-certainty evidence).

##### Secondary Outcomes

(1) Adverse drug events

Two RCTs with 971 patients (fosfomycin *n* = 509, FQ *n* = 462) reported adverse drug events [30,31]. There is probably little to no difference in the incidence of drug adverse events between fosfomycin and FQ (RR 0.87, 95% CI 0.37 to 2.06; I^2^ = not applicable; moderate-certainty evidence). We downgraded the certainty of the evidence for serious study limitations (−1).

One NRS with 1109 patients (fosfomycin *n* = 632, FQ *n* = 477) reported adverse drug events [6]. Fosfomycin may result in little to no difference in drug adverse events compared to FQ (RR 1.51, 95% CI 0.28 to 8.21; I^2^ = not applicable; low-certainty evidence).

Based on the evidence from the RCTs that provided evidence of higher certainty, there is probably little to no difference in drug adverse events between fosfomycin and FQ (moderate-certainty evidence).

(2) Positive urine cultures

Three RCTs with 1175 patients (fosfomycin *n* = 611, FQ *n* = 564) reported positive urine cultures [30,31,33]. Fosfomycin may reduce positive urine cultures slightly compared with FQ (RR 0.57, 95% CI 0.30 to 1.08; I^2^ = 0%; low-certainty evidence). We downgraded the certainty of the evidence for serious study limitations (−1) and imprecision (−1).

Three NRSs with 1926 patients (fosfomycin *n* = 817, FQ *n* = 1109) reported positive urine cultures [6,17,34]. We are very uncertain whether fosfomycin results in fewer positive urine cultures than FQ (RR 0.28, 95% CI 0.08 to 1.06; I^2^ = 72%; very low-certainty evidence). We downgraded the certainty of the evidence for inconsistency (−1) and imprecision (−1).

Based on the evidence from the RCTs that provided evidence of higher certainty, fosfomycin may reduce positive urine culture incidence slightly compared with FQ (low-certainty evidence).

(3) Positive blood cultures

One RCT with 204 patients (fosfomycin *n* = 102, FQ *n* = 102) reported positive blood cultures [33]. Fosfomycin may result in little to no difference in positive blood cultures compared with FQ (RR 5.00, 95% CI 0.24 to 102.8; I^2^ = not applicable; low-certainty evidence). We downgraded the certainty of the evidence for serious study limitations (−1) and imprecision (−1).

Three NRSs with 2316 patients (fosfomycin *n* = 1150, FQ *n* = 1166) reported positive blood cultures [6,34,35]. We are very uncertain whether fosfomycin resulted in fewer positive blood cultures than FQ (RR 0.26, 95% CI 0.06 to 1.15; I^2^ = 49%; very low-certainty evidence). We downgraded the certainty of the evidence for inconsistency (−1) and imprecision (−1).

Based on the evidence from the RCTs that provided evidence of higher certainty, Fosfomycin may result in little to no difference in positive blood cultures compared with FQ (low-certainty evidence).

(4) FQ resistance

Three RCTs with 1175 patients (fosfomycin *n* = 611, FQ *n* = 564) reported FQ resistance [30,31,33]. Fosfomycin may have slightly less FQ resistance compared with FQ (RR 0.30, 95% CI 0.11 to 0.79; I^2^ = 0%; low-certainty evidence). We downgraded the certainty of the evidence for serious study limitations (−1) and imprecision (−1).

Three NRSs with 1926 patients (fosfomycin *n* = 817, FQ *n* = 1109) reported FQ resistance [6,34,35]. Fosfomycin may result in little to no difference in FQ resistance compared with FQ (RR 0.29, 95% CI 0.13 to 0.69; I^2^ = 0%; low-certainty evidence).

Based on the evidence from the RCTs and NRSs, fosfomycin may have slightly less FQ resistance compared to FQ (low-certainty evidence).

#### 3.4.2. Fosfomycin and FQ versus FQ

Please refer to Table 2 and Supplementary Figures S8–S11. Two NRSs with 3855 patients (fosfomycin + FQ *n* = 1531, FQ *n* = 2324) were included in the analysis [38,39].

##### Primary Outcomes

(1) Febrile UTI

Fosfomycin combined with FQ may result in little to no difference in febrile UTIs compared with FQ (RR 0.13, 95% CI 0.04 to 0.43; I^2^ = 0%; low-certainty evidence).

(2) Afebrile UTI, (3) Overall UTI

We did not find any studies reporting these outcomes.

##### Secondary Outcomes

(1) Adverse drug events

We did not find any studies reporting this outcome.

(2) Positive urine cultures

Fosfomycin combined with FQ may result in little to no difference in positive urine cultures compared with FQ (RR 0.17, 95% CI 0.04 to 0.71; I^2^ = 0%; low-certainty evidence).

(3) Positive blood cultures

Fosfomycin combined with FQ may result in little to no difference in positive blood cultures compared with FQ (RR 0.07, 95% CI 0.01 to 0.37; I^2^ = 0%; low-certainty evidence).

(4) FQ resistance

Fosfomycin combined with FQ may result in little to no difference in fluoroquinolone resistance compared with FQ (RR 0.12, 95% CI 0.02 to 0.86; I^2^ = 24%; low-certainty evidence).

For the other comparisons (3. Fosfomycin versus β-lactam or FQ [37]; 4. Fosfomycin versus FQ and metronidazole [15]; 5. Fosfomycin versus FQ and metronidazole and gentamycin [36]), please see Appendix A and Supplementary Figures S12–S19.

## 4. Discussion

### 4.1. Main Findings

A systematic review, including six RCTs [15,16,30,31,32,33] and eight NRSs [6,17,34,35,36,37,38,39], assessing the prophylactic effect of fosfomycin on UTIs after TRUS biopsies was conducted. Of the five comparisons, the primary outcomes of the two most clinically significant comparisons were the primary focus. For fosfomycin alone, in five RCTs [16,30,31,32,33], moderate-certainty evidence indicated that there was probably little to no difference in febrile UTI incidence compared with FQ. For fosfomycin in combination with FQ, in two NRSs [38,39], there may be little to no difference in febrile UTIs compared with FQ but with low-certainty evidence.

### 4.2. Relation to Previous Works

There were four previous systematic reviews [7,18,19,20] on the same topic; however, none have the same rigorous methodology as this study. These previous reviews only presented relative effect size measure, which interprets statistical significance rather than clinically meaningful differences.

A systematic review by Noreikaite et al. [18] included three RCTs and two NRSs comparing fosfomycin versus non-fosfomycin antimicrobial prophylaxis for TRUS biopsies and involved a total of 3112 patients. This review suggests that fosfomycin has significantly lower overall UTIs (RR 0.20, 95% CI 0.13 to 0.30) and febrile UTIs (RR 0.27, 95% CI 0.16 to 0.45) in comparison with a quinolone-based prophylaxis regimen for TRUS biopsies. Regarding the adverse effects, their results were similar to ours, with around 1% (14 of 1343) of the patients developing minor adverse side effects in the fosfomycin cohort, confirming its safety. However, since this review meta-analyzed the RCTs and NRSs together, only investigated the study quality of the RCTs, and rated the certainty of the evidence of the pooled effect from the RCTS with NRSs, the results are questionable.

Roberts et al. published a study that meta-analyzed three RCTs and two NRSs comparing fosfomycin with FQ prophylaxis to prevent TRUS biopsy-related infectious complications and contained 3112 patients using individual participant data [7]. The authors reported that fosfomycin was more effective as a TRUS biopsy prophylaxis than FQ (OR 0.22, 95% CI 0.09 to 0.54). When the results were subdivided by complication severity grade, a greater infection odds reduction was seen across all grades for the fosfomycin-treated patients but more so for the higher-grade infectious complications: Grade 2 (Bacteremia, febrile UTI, or urosepsis; OR 0.13, 95% CI 0.07 to 0.26) versus Grade 1 infectious complications (Bacteriuria and afebrile UTI; OR 0.30, 95% CI 0.13 to 0.69). Moreover, fosfomycin reduced infectious complications regardless of FQ resistance or sensitive status. However, this study also pooled the RCT and NRS results, and looking at the pooled RCT results revealed no difference in overall complications compared with FQ. The study did not provide certainty of the evidence.

Freitas et al. published a study that included two RCTs and two NRSs with 2331 males [19]. In this study, fosfomycin prophylaxis resulted in significantly fewer afebrile (OR 0.21, 95% CI 0.12 to 0.38) and febrile (OR 0.15, 95% CI 0.07 to 0.31) UTIs than ciprofloxacin. The study also assessed study quality using the Cochrane Risk of Bias Tool for RCTs and the Newcastle-Ottawa Scale for observational studies. However, it did not provide certainty of the evidence. Furthermore, this study also combined the RCT and NRS estimates.

The most recently published systematic review by Pilatz et al. stands out favorably for its reliable methodologic approach [20]. Overall, 59 RCTs and seven different antimicrobial interventions were included, and a subgroup analysis of three studies with 1239 participants comparing fosfomycin versus standard FQs was done. They concluded that fosfomycin was an alternative to FQ with reduced rates of infectious complications (RR 0.49, 95% CI 0.27 to 0.87). However, the certainty of the evidence indicated very low certainty. Additionally, this review was limited due to fewer studies comparing fosfomycin with FQ (compared to against five RCTs in ours).

We found no systematic reviews that compared fosfomycin and FQ combined with FQ or fosfomycin with other antibiotic regimens. In summary, current existing systematic reviews advocate a protective effect of fosfomycin on infectious complications after a TRUS biopsy compared with FQ. This result differed from our study, where fosfomycin had a similar effect on infectious complications after a TRUS biopsy compared with FQ. Since previous systematic reviews combined the RCT and NRS point estimates, the meta-estimate is weighted toward the results of the NRSs [7,40]. However, fosfomycin combined with FQ also appears to have a similar effect on febrile UTIs compared with FQ.

### 4.3. Strength and Limitations

The merit point of the present study is its rigorous methodology, which includes a prospectively registered written protocol, a comprehensive literature search developed and executed by an experienced information specialist, study selection, data abstraction, certainty of the evidence rating using GRADE independently and in duplicate, and a contextualized interpretation of the outcomes considering relative and absolute effect size estimates. Moreover, existing systematic reviews did not compare fosfomycin and other antibiotic regimens or fosfomycin combined with FQ versus FQ.

However, this study has several drawbacks. Individual studies in the review were heterogeneous regarding the fosfomycin regimen (single dose or two doses), targeted prophylaxis with rectal cultures, and geographic locations. These potentially had significant implications on patient outcomes. A recent study confirmed the ability of fosfomycin to distribute to the prostate and seminal vesicles after one single dose and that a two-consecutive-dose regimen increases antibiotic availability inside these peripheral tissues [41]. Establishing a standardized fosfomycin regimen for TRUS biopsies will be a topic of future research. We may have lost some articles inadvertently when doing the literature search, and our study may not be the latest at the time of publication. Different conclusions can be reached depending on the “clinically significant” threshold. Finally, we could not perform predefined subgroups and sensitivity analyses due to the scarcity of the data and the few trials that were included in each comparison.

Nevertheless, this study provides a more accurate analysis for physicians to assist them in better managing their patients. In particular, this is the first systematic review that registered fosfomycin alone and fosfomycin combined with FQ for the intervention group.

#### Implications

The European Association of Urology guidelines on managing prostate biopsies to reduce infectious complications recommend that transperineal biopsies be the first choice. If not feasible, a transrectal biopsy should be considered as a second choice. If FQ is not licensed, alternative antibiotics such as fosfomycin (3 g before and 3 g 24–48 h after the biopsy) were considered with very low certainty [42]. Moreover, due to a negative benefit-risk balance, the European Commission has recently restricted FQ for antibiotic prophylaxis in urological operations and diagnostic interventions [43].

Considering the increase in FQ resistance of fecal flora and uropathogens [2,11,44] and high sensitivity to fosfomycin of uropathogens [44], this study provides the current best evidence when it comes to decision-making about antibiotic prophylaxis before a TRUS biopsy. Based on the moderate to low certainty of the evidence, it is important to emphasize that fosfomycin was not inferior to FQ in prophylactic effect on UTIs after a TRUS biopsy. Urologists should check their local guidance about using fosfomycin for prostate biopsies. Decision-making should consider other conveniences, such as patient compliance and cost-effectiveness. Since fosfomycin is a helpful agent used in variable infections, careful use is recommended to reduce the development of resistance similar to what is observed for FQ. Thus, local microbiological surveillance protocols and resistance patterns should be performed by the treating clinicians.

Transperineal prostate biopsies, magnetic resonance imaging fusion prostate biopsies [45], and targeted antibiotic prophylaxis using rectal swabs are ways to reduce infectious complications after a TRUS biopsy. Appropriate antibiotic prophylaxis with fosfomycin will require some of these strategies. This topic should be better informed by future research.

## 5. Conclusions

Compared with FQ, fosfomycin or fosfomycin and FQ combined had a similar prophylactic effect on UTIs after a transrectal prostate biopsy. The certainty of the evidence for the primary outcomes of this review was moderate to low, which matches our confidence in the reported effect estimates, which are likely to be close to the true effect or are limited. Regardless of which regimen is adopted in clinical practice, the fosfomycin course was short (one or two doses only). Hence, it is likely to have good patient compliance, and decreased UTIs and admissions to the hospital may reduce overall costs. Given the increasing FQ resistance and its ease to use with a relatively safe profile, fosfomycin may be a good option for antibiotic prophylaxis.

## Figures and Tables

**Table 1:** Fosfomycin compared to fluoroquinolone for antibiotic prophylaxis in men undergoing transrectal prostate biopsy.

**Patient or population:** men undergoing transrectal prostate biopsy **Setting:** RCTs and NRSs (single and multi-centers)/Italy, Philippines, US, France, Belgium, Turkey, Spain **Intervention:** Fosfomycin **Comparison:** Fluoroquinolone						
**Outcomes ^a^**	**№ of Participants** **(Studies)**	**Certainty of Evidence** **(GRADE)**	**Relative Effect** **(95% CI)**	**Anticipated Absolute Effects**		**What Happens?**
				**Risk with Fluoroquinolone**	Risk Difference with Fosfomycin *	
**Febrile UTI** Follow-up: range 2 to 4 weeks MCID: absolute 2% reduction/increase	1511 (5 RCTs)	⨁⨁⨁◯ Moderate ^b^	**RR 0.84** (0.42 to 1.69)	23 per 1000	**4 fewer per 1000** (14 fewer to 16 more)	There is probably little to no difference in febrile UTI incidence between fosfomycin and fluoroquinolone.
**Afebrile UTI** Follow-up: range 2 to 4 weeks MCID: absolute 5% reduction/increase	1511 (5 RCTs)	⨁⨁⨁◯ Moderate ^b^	**RR 0.43** (0.23 to 0.78)	51 per 1000	**29 fewer per 1000** (39 fewer to 11 fewer)	There is probably little to no difference in afebrile UTI incidence between fosfomycin and fluoroquinolone.
**Overall UTI** Follow-up: range 2 to 4 weeks MCID: absolute 5% reduction/increase	1511 (5 RCTs)	⨁⨁⨁◯ Moderate ^b^	**RR 0.53** (0.31 to 0.92)	74 per 1000	**35 fewer per 1000** (51 fewer to 6 fewer)	There is probably little to no difference in overall UTI incidence between fosfomycin and fluoroquinolone.
**Drug adverse events** Follow-up: range 2 to 4 weeks MCID: absolute 5% reduction/increase	971 (2 RCTs)	⨁⨁⨁◯ Moderate ^b^	**RR 0.87** (0.37 to 2.06)	22 per 1000	**3 fewer per 1000** (14 fewer to 23 more)	There is probably little to no difference in drug adverse events between fosfomycin and fluoroquinolone.
**Positive urine culture** Follow-up: range 2 to 4 weeks MCID: absolute 2% reduction/increase	1175 (3 RCTs)	⨁⨁◯◯ Low ^b,c^	**RR 0.57** (0.30 to 1.08)	46 per 1000	**20 fewer per 1000** (32 fewer to 4 more)	Fosfomycin may have less positive urine culture slightly compared to fluoroquinolone.
**Positive blood culture** Follow-up: range 2 to 4 weeks MCID: absolute 2% reduction/increase	204 (1 RCT)	⨁⨁◯◯ Low ^d,e^	**RR 5.00** (0.24 to 102.87)	-	**-**	Fosfomycin may result in little to no difference in positive blood culture compared to fluoroquinolone.
**Floroquinolone resistance** Follow-up: range 2 to 4 weeks MCID: absolute 2% reduction/increase	1175 (3 RCTs)	⨁⨁◯◯ Low ^b,c^	**RR 0.30** (0.11 to 0.79)	32 per 1000	**22 fewer per 1000** (28 fewer to 7 fewer)	Fosfomycin may have less fluoroquinolone resistance slightly compared to fluoroquinolone.
* **The risk in the intervention group** (and its 95% confidence interval) is based on the assumed risk in the comparison group and the **relative effect** of the intervention (and its 95% CI). **CI:** confidence interval; **MCID**: minimal clinically important difference; **NRS**: non-randomized study; **RCT:** randomized controlled trial; **RR:** risk ratio; **UTI:** urinary tract infection						
**GRADE Working Group grades of evidence** **High certainty:** we are very confident that the true effect lies close to that of the estimate of the effect. **Moderate certainty:** we are moderately confident in the effect estimate: the true effect is likely to be close to the estimate of the effect, but there is a possibility that it is substantially different. **Low certainty:** our confidence in the effect estimate is limited: the true effect may be substantially different from the estimate of the effect. **Very low certainty:** we have very little confidence in the effect estimate: the true effect is likely to be substantially different from the estimate of effect.						

**Explanations: **^a^. Certainty of evidence of RCTs was higher than NRSs. ^b^. Downgraded for study limitations: RCTs, some concerns, or high risk of overall bias in the included studies (−1). ^c^. Downgraded one level for imprecision: confidence interval crossed assumed of a clinically important difference. ^d^. Downgraded for study limitations: RCTs, some concerns of overall bias in the included studies (−1). ^e^. Downgraded one level for imprecision: There is no event in the control group.

**Table 2:** Fosfomycin and fluoroquinolone compared to fluoroquinolone for the antibiotic prophylaxis in men undergoing transrectal prostate biopsy.

**Patient or population:** men undergoing transrectal prostate biopsy **Setting:** NRS/single center (South Korea), multi-center (Canada) **Intervention:** Fosfomycin and fluoroquinolone **Comparison:** fluoroquinolone						
**Outcomes**	**№ of Participants** **(Studies)**	**Certainty ** **of Evidence** **(GRADE)**	**Relative Effect** **(95% CI)**	**Anticipated Absolute Effects**		**What Happens?**
				**Risk with Fluoroquinolone**	Risk Difference with Fosfomycin + Fluoroquinolone *	
**Febrile UTI** follow-up: 4 weeks MCID: absolute 2% reduction/increase	3855 (2 NRSs)	⨁⨁◯◯ Low	**RR 0.13** (0.04 to 0.43)	19 per 1000	**16 fewer per 1000** (18 fewer to 11 fewer)	There may be little to no difference in febrile UTI incidence between fosfomycin and fluoroquinolone and fluoroquinolone.
**Afebrile UTI** Not reported						We did not find any studies reporting this outcome.
**Overall UTI** Not reported						We did not find any studies reporting this outcome.
**Drug adverse events** Not reported						We did not find any studies reporting this outcome.
**Positive urine culture** follow-up: 4 weeks MCID: absolute 2% reduction/increase	3855 (2 NRSs)	⨁⨁◯◯ Low	**RR 0.17** (0.04 to 0.71)	9 per 1000	**8 fewer per 1000** (9 fewer to 3 fewer)	There may be little to no difference in positive urine culture between fosfomycin and fluoroquinolone and fluoroquinolone.
**Positive blood culture** follow-up: 4 weeks MCID: absolute 2% reduction/increase	3855 (2 NRSs)	⨁⨁◯◯ Low	**RR 0.07** (0.01 to 0.37)	13 per 1000	**12 fewer per 1000** (13 fewer to 8 fewer)	There may be little to no difference in positive blood culture between fosfomycin and fluoroquinolone and fluoroquinolone.
**Floroquinolone resistance** follow-up: 4 weeks MCID: absolute 2% reduction/increase	3855 (2 NRSs)	⨁⨁◯◯ Low	**RR 0.12** (0.02 to 0.86)	11 per 1000	**10 fewer per 1000** (11 fewer to 2 fewer)	There may be little to no difference in fluoroquinolone resistance between fosfomycin and fluoroquinolone and fluoroquinolone.
* **The risk in the intervention group** (and its 95% confidence interval) is based on the assumed risk in the comparison group and the **relative effect** of the intervention (and its 95% CI). **CI:** confidence interval; **MCID**: minimal clinically important difference; **NRS**: non-randomized study; **RR:** risk ratio; **UTI:** urinary tract infection						
**GRADE Working Group grades of evidence** **High certainty:** we are very confident that the true effect lies close to that of the estimate of the effect. **Moderate certainty:** we are moderately confident in the effect estimate: the true effect is likely to be close to the estimate of the effect, but there is a possibility that it is substantially different. **Low certainty:** our confidence in the effect estimate is limited: the true effect may be substantially different from the estimate of the effect. **Very low certainty:** we have very little confidence in the effect estimate: the true effect is likely to be substantially different from the estimate of effect.						

## Data Availability

The dataset collected and analyzed in the present study is obtainable from the corresponding author on reasonable request.

## Supplementary Materials

The following supporting information can be downloaded at: https://www.mdpi.com/article/10.3390/medicina59050911/s1 [46,47,48,49,50].

## Appendix A

**Table A1:** The quality assessment included non-randomized studies by Newcastle-Ottawa Scale.

Study	Selection				Comparability	Outcome			Total Selection	Total Comparability	Total Outcome	Quality
	Representativeness of the Exposed Cohort	Selection of the Non-Exposed cohort	Ascertainment of Exposure	Demonstration That Outcome of Interest was Not Present at Start of Study	Comparability of Cohorts Based on Design or Analysis	Assessment of Outcome	Followup long Enough for Outcome to Occur	Adequacy of Follow up of Cohorts				
**Lim 2021** [39]	1	1	1	1	1	1	1	1	4	1	3	Good
**Cai 2017** [6]	1	1	1	1	2	1	1	1	4	2	3	Good
**Cimino 2020** [37]	1	1	1	1	1	1	1	1	4	1	3	Good
**Colhoun 2015** [35]	1	1	1	1	1	1	1	1	4	1	3	Good
**Delory 2021** [17]	1	1	1	1	1	0	1	1	4	1	2	Good
**Morin 2020** [38]	1	1	1	1	1	1	1	1	4	1	3	Good
**Ongun 2012** [34]	1	1	1	1	2	1	1	1	4	2	3	Good
**Yang 2019** [36]	1	1	0	0	1	0	1	1	2	1	2	Moderate

**Table A2:** Certainty of evidence decisions (Fosfomycin versus fluoroquinolone).

Outcomes	Study Design	Certainty of Evidence(GRADE)
Febrile UTI	RCT	Moderate
	NRS	Very low
Afebrile UTI	RCT	Moderate
	NRS	Very low
Overall UTI	RCT	Moderate
	NRS	Very low
Drug adverse events	RCT	Moderate
	NRS	Low
Positive urine culture	RCT	Low
	NRS	Very low
Positive blood culture	RCT	Low
	NRS	Very low
Floroquinolone resistance	RCT	Low
	NRS	Low

Other comparisons

3. Fosfomycin versus β-Lactam or FQ

One NRS with 516 patients (fosfomycin n = 258, β-lactam, or FQ n = 258) was included in the analysis [37].

*Primary outcomes*

(1) Febrile UTI

We are very uncertain about the effect of fosfomycin on febrile UTI compared to β-lactam or FQ (RR 0.79, 95% CI 0.63 to 0.98; I^2^ = not applicable; very low-certainty evidence). We downgraded the certainty of evidence for imprecision (–1).

(2) Afebrile UTI, 3) Overall UTI

We did not find any studies reporting these outcomes.

*Secondary outcomes*

(1) Drug adverse events; (2) Positive urine culture; (3) Positive blood culture; (4) FQ resistance.

We did not find any studies reporting these outcomes.

4. Fosfomycin versus FQ and Metronidazole

One RCT with 412 patients (fosfomycin n = 202, FQ + metronidazole n = 210) was included in the analysis [15].

*Primary outcomes*

(1) Febrile UTI

Fosfomycin may result in little to no difference in febrile UTI compared to FQ + metronidazole (RR 0.26, 95% CI 0.03 to 2.31; I^2^ = not applicable; low-certainty evidence). We downgraded the certainty of evidence for serious study limitations (–1) and imprecision (–1).

(2) Afebrile UTI

Fosfomycin may reduce afebrile UTI slightly compared to FQ + metronidazole (RR 0.22, 95% CI 0.06 to 0.76; I^2^ = not applicable; low-certainty evidence). We downgraded the certainty of evidence for serious study limitations (–1) and imprecision (–1).

(3) Overall UTI

Fosfomycin may reduce overall UTI slightly compared to FQ + metronidazole (RR 0.23, 95% CI 0.08 to 0.67; I^2^ = not applicable; low-certainty evidence). We downgraded the certainty of evidence for serious study limitations (–1) and imprecision (–1).

*Secondary outcomes*

(1) Drug adverse events

Fosfomycin likely results in little to no difference in drug adverse events compared to FQ + metronidazole (RR 0.26, 95% CI 0.03 to 2.31; I^2^ = not applicable; moderate-certainty evidence). We downgraded the certainty of evidence for serious study limitations (–1).

(2) Positive urine culture

Fosfomycin may result in little to no difference in positive urine culture compared to FQ + metronidazole (RR 0.26, 95% CI 0.03 to 2.31; I^2^ = not applicable; low-certainty evidence). We downgraded the certainty of evidence for serious study limitations (–1) and imprecision (–1).

(3) Positive blood culture

We are very uncertain about the effect of fosfomycin on positive blood culture compared to FQ + metronidazole. There are no events in either arm.

(4) Fluoroquinolone resistance.

Fosfomycin may have less fluoroquinolone resistance slightly compared to FQ + metronidazole (RR 0.24, 95% CI 0.07 to 0.83; I^2^ = not applicable; low-certainty evidence). We downgraded the certainty of evidence for serious study limitations (–1) and imprecision (–1).

5. Fosfomycin versus FQ and Metronidazole and Gentamycin

One NRS with 171 patients (fosfomycin n = 89, FQ + metronidazole + gentamycin n = 82) was included in the analysis [36].

*Primary outcomes*

(1) Febrile UTI; (2) Afebrile UTI

We did not find any studies reporting these outcomes.

(3) Overall UTI

We are very uncertain about the effect of fosfomycin on overall UTI (RR 0.46, 95% CI 0.04 to 4.99; I^2^ = not applicable; very low-certainty evidence). We downgraded the certainty of evidence for serious study limitations (–1) and imprecision (–1).

*Secondary outcomes*

(1) Drug adverse events; (2) Positive urine culture; (3) Positive blood culture; (4) FQ resistance.

We did not find any studies reporting these outcomes.

**Table A3:** Fosfomycin compared to β-lactam or fluoroquinolone for the antibiotic prophylaxis in men undergoing transrectal prostate biopsy.

**Patient or population:** men undergoing transrectal prostate biopsy **Setting:** NRS/multicenter (Italy) **Intervention:** Fosfomycin **Comparison:** β-lactam or fluoroquinolone						
**Outcomes**	**№ of Participants** **(Studies)**	**Certainty ** **of Evidence** **(GRADE)**	**Relative Effect** **(95% CI)**	**Anticipated Absolute Effects**		**What Happens?**
				**Risk with β-lactam or Fluoroquinolone**	**Risk Difference with Fosfomycin + Fluoroquinolone** *****	
**Febrile UTI** follow-up: 4 weeks MCID: absolute 2% reduction/increase	516 (1 NRS)	⨁◯◯◯ Very low ^a^	**RR 0.79** (0.63 to 0.98)	434 per 1000	91 fewer per 1000 (161 fewer to 9 fewer)	We are very uncertain about the effect of fosfomycin on febrile UTI.
**Afebrile UTI** Not reported						We did not find any studies reporting this outcome.
**Overall UTI** Not reported						We did not find any studies reporting this outcome.
**Drug adverse events** Not reported						We did not find any studies reporting this outcome.
**Positive urine culture** Not reported						We did not find any studies reporting this outcome.
**Positive blood culture** Not reported						We did not find any studies reporting this outcome.
**Floroquinolone resistance** Not reported						We did not find any studies reporting this outcome.
* **The risk in the intervention group** (and its 95% confidence interval) is based on the assumed risk in the comparison group and the **relative effect** of the intervention (and its 95% CI). **CI:** confidence interval; **MCID**: minimal clinically important difference; **NRS**: non-randomized study; **RR:** risk ratio; **UTI:** urinary tract infection						
**GRADE Working Group grades of evidence** **High certainty:** we are very confident that the true effect lies close to that of the estimate of the effect. **Moderate certainty:** we are moderately confident in the effect estimate: the true effect is likely to be close to the estimate of the effect, but there is a possibility that it is substantially different. **Low certainty:** our confidence in the effect estimate is limited: the true effect may be substantially different from the estimate of the effect. **Very low certainty:** we have very little confidence in the effect estimate: the true effect is likely to be substantially different from the estimate of effect.						

**Explanations**: ^a^. Downgraded one level for imprecision: confidence interval crossed assumed of a clinically important difference.

**Table A4:** Fosfomycin compared to fluoroquinolone and metronidazole for antibiotic prophylaxis in men undergoing transrectal prostate biopsy.

**Patient or population:** men undergoing transrectal prostate biopsy **Setting:** RCTs (single center)/Egypt **Intervention:** Fosfomycin **Comparison:** fluoroquinolone and metronidazole						
**Outcomes ^a^**	**№ of Participants** **(Studies)**	**Certainty of Evidence** **(GRADE)**	**Relative Effect** **(95% CI)**	**Anticipated Absolute Effects**		**What Happens?**
				**Risk with Fluoroquinolone and Metronidazole**	**Risk Difference with Fosfomycin** *****	
**Febrile UTI** Follow-up: range 2 to 4 weeks MCID: absolute 2% reduction/increase	412 (1 RCT)	⨁⨁◯◯ Low ^a,b^	**RR 0.26** (0.03 to 2.31)	19 per 1000	14 fewer per 1000 (18 fewer to 25 more)	Fosfomycin may result in little to no difference in febrile UTI compared to fluoroquinolone and metronidazole.
**Afebrile UTI** Follow-up: range 2 to 4 weeks MCID: absolute 5% reduction/increase	412 (1 RCT)	⨁⨁◯◯ Low ^a,b^	**RR 0.22** (0.06 to 0.76)	67 per 1000	52 fewer per 1000 (63 fewer to 16 fewer)	Fosfomycin may reduce afebrile UTI slightly compared to fluoroquinolone and metronidazole.
**Overall UTI** Follow-up: range 2 to 4 weeks MCID: absolute 5% reduction/increase	412 (1 RCT)	⨁⨁◯◯ Low ^a,b^	**RR 0.23** (0.08 to 0.67)	86 per 1000	66 fewer per 1000 (79 fewer to 28 fewer)	Fosfomycin may reduce overall UTI slightly compared to fluoroquinolone and metronidazole.
**Drug adverse events** Follow-up: range 2 to 4 weeks MCID: absolute 5% reduction/increase	412 (1 RCT)	⨁⨁⨁◯ Moderate ^a^	**RR 0.26** (0.03 to 2.31)	19 per 1000	14 fewer per 1000 (18 fewer to 25 more)	There is probably little to no difference in drug adverse events between fosfomycin and fluoroquinolone and metronidazole.
**Positive urine culture** Follow-up: range 2 to 4 weeks MCID: absolute 2% reduction/increase	412 (1 RCT)	⨁⨁◯◯ Low ^a,b^	**RR 0.26** (0.03 to 2.31)	19 per 1000	14 fewer per 1000 (18 fewer to 25 more)	There may be little to no difference in positive urine culture between fosfomycin and fluoroquinolone and metronidazole.
**Positive blood culture** Follow-up: range 2 to 4 weeks MCID: absolute 2% reduction/increase	412 (1 RCT)	⨁◯◯◯ Very low ^a,c^	not estimable			We are very uncertain about the effect of fosfomycin on positive blood culture compared to fluoroquinolone and metronidazole.
**Floroquinolone resistance** Follow-up: range 2 to 4 weeks MCID: absolute 2% reduction/increase	412 (1 RCT)	⨁⨁◯◯ Low ^a,b^	**RR 0.24** (0.07 to 0.83)	62 per 1000	47 fewer per 1000 (58 fewer to 11 fewer)	Fosfomycin may have less fluoroquinolone resistance slightly compared to fluoroquinolone and metronidazole.
* **The risk in the intervention group** (and its 95% confidence interval) is based on the assumed risk in the comparison group and the **relative effect** of the intervention (and its 95% CI). **CI:** confidence interval; **MCID**: minimal clinically important difference; **NRS**: non-randomized study; **RCT:** randomized controlled trial; **RR:** risk ratio; **UTI:** urinary tract infection						
**GRADE Working Group grades of evidence** **High certainty:** we are very confident that the true effect lies close to that of the estimate of the effect. **Moderate certainty:** we are moderately confident in the effect estimate: the true effect is likely to be close to the estimate of the effect, but there is a possibility that it is substantially different. **Low certainty:** our confidence in the effect estimate is limited: the true effect may be substantially different from the estimate of the effect. **Very low certainty:** we have very little confidence in the effect estimate: the true effect is likely to be substantially different from the estimate of effect.						

**Explanations**: ^a^. Downgraded for study limitations: RCTs, some concerns of overall bias in the included studies (–1). ^b^. Downgraded one level for imprecision: confidence interval crossed assumed of a clinically important difference. ^c^. Downgraded two levels for imprecision: There are no events in either arm.

**Table A5:** Fosfomycin compared to fluoroquinolone and metronidazole and gentamycin for antibiotic prophylaxis in men undergoing transrectal prostate biopsy.

**Patient or population:** men undergoing transrectal prostate biopsy **Setting:** NRS/single center (England) **Intervention:** Fosfomycin **Comparison:** fluoroquinolone and metronidazole and gentamycin						
**Outcomes**	**№ of Participants** **(Studies)**	**Certainty ** **of Evidence** **(GRADE)**	**Relative Effect (95% CI)**	**Anticipated Absolute Effects**		**What Happens?**
				**Risk with Fluoroquinolone and Metronidazole and Gentamycin**	**Risk Difference with Fosfomycin** *****	
**Febrile UTI** Not reported						We did not find any studies reporting this outcome
**Afebrile UTI** Not reported						We did not find any studies reporting this outcome.
**Overall UTI** follow-up: 4 weeks MCID: absolute 5% reduction/increase	171 (1 NRS)	⨁◯◯◯ Very low ^a,b^	**RR 0.46** (0.04 to 4.99)	24 per 1000	13 fewer per 1000 (23 fewer to 97 more)	We are very uncertain about the effect of fosfomycin on overall UTI.
**Drug adverse events** Not reported						We did not find any studies reporting this outcome.
**Positive urine culture** Not reported						We did not find any studies reporting this outcome.
**Positive blood culture** Not reported						We did not find any studies reporting this outcome.
**Floroquinolone resistance** Not reported						We did not find any studies reporting this outcome.
* **The risk in the intervention group** (and its 95% confidence interval) is based on the assumed risk in the comparison group and the **relative effect** of the intervention (and its 95% CI). **CI:** confidence interval; **MCID**: minimal clinically important difference; **NRS**: non-randomized study; **RR:** risk ratio; **UTI:** urinary tract infection						
**GRADE Working Group grades of evidence** **High certainty:** we are very confident that the true effect lies close to that of the estimate of the effect. **Moderate certainty:** we are moderately confident in the effect estimate: the true effect is likely to be close to the estimate of the effect, but there is a possibility that it is substantially different. **Low certainty:** our confidence in the effect estimate is limited: the true effect may be substantially different from the estimate of the effect. **Very low certainty:** we have very little confidence in the effect estimate: the true effect is likely to be substantially different from the estimate of effect.						

**Explanations**: ^a^. Downgraded for study limitations: NRS; moderate quality. ^b^. Downgraded one level for imprecision: confidence interval crossed assumed of a clinically important difference.
