# Supplementary material for: Fosfomycin for Antibiotic Prophylaxis in Men Undergoing a Transrectal Prostate Biopsy: A Systematic Review and Meta-Analysis
Source: Medicina (Kaunas). 2023 May 10;59(5):911. doi: 10.3390/medicina59050911 (PMC10221023; doi:10.3390/medicina59050911)
Supplement: Supplementary file 1 [file medicina-59-00911-s001.zip › Supplement figures.pptx]

## Slide 1
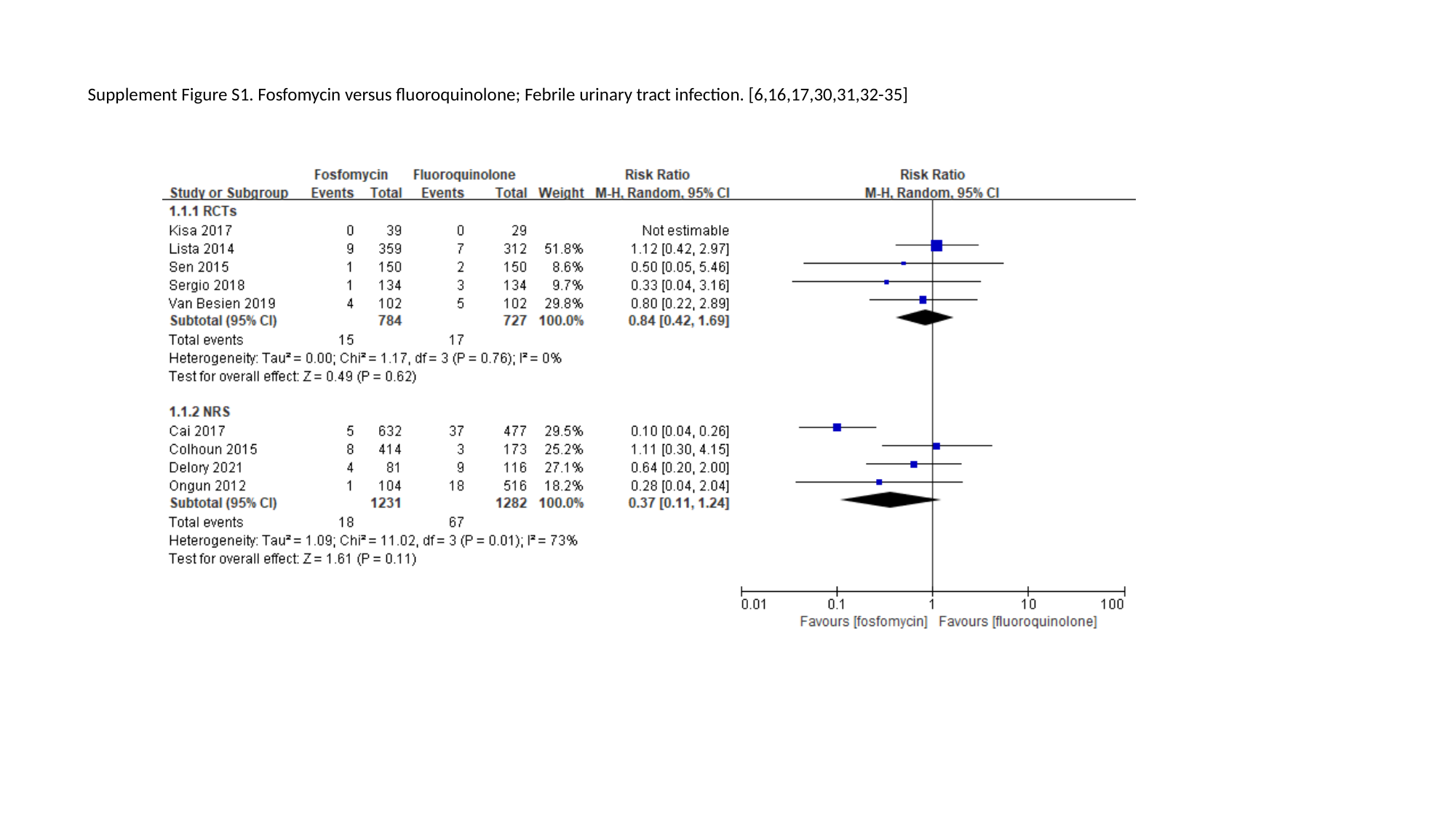

Supplement Figure S1. Fosfomycin versus fluoroquinolone; Febrile urinary tract infection. [6,16,17,30,31,32-35]

## Slide 2
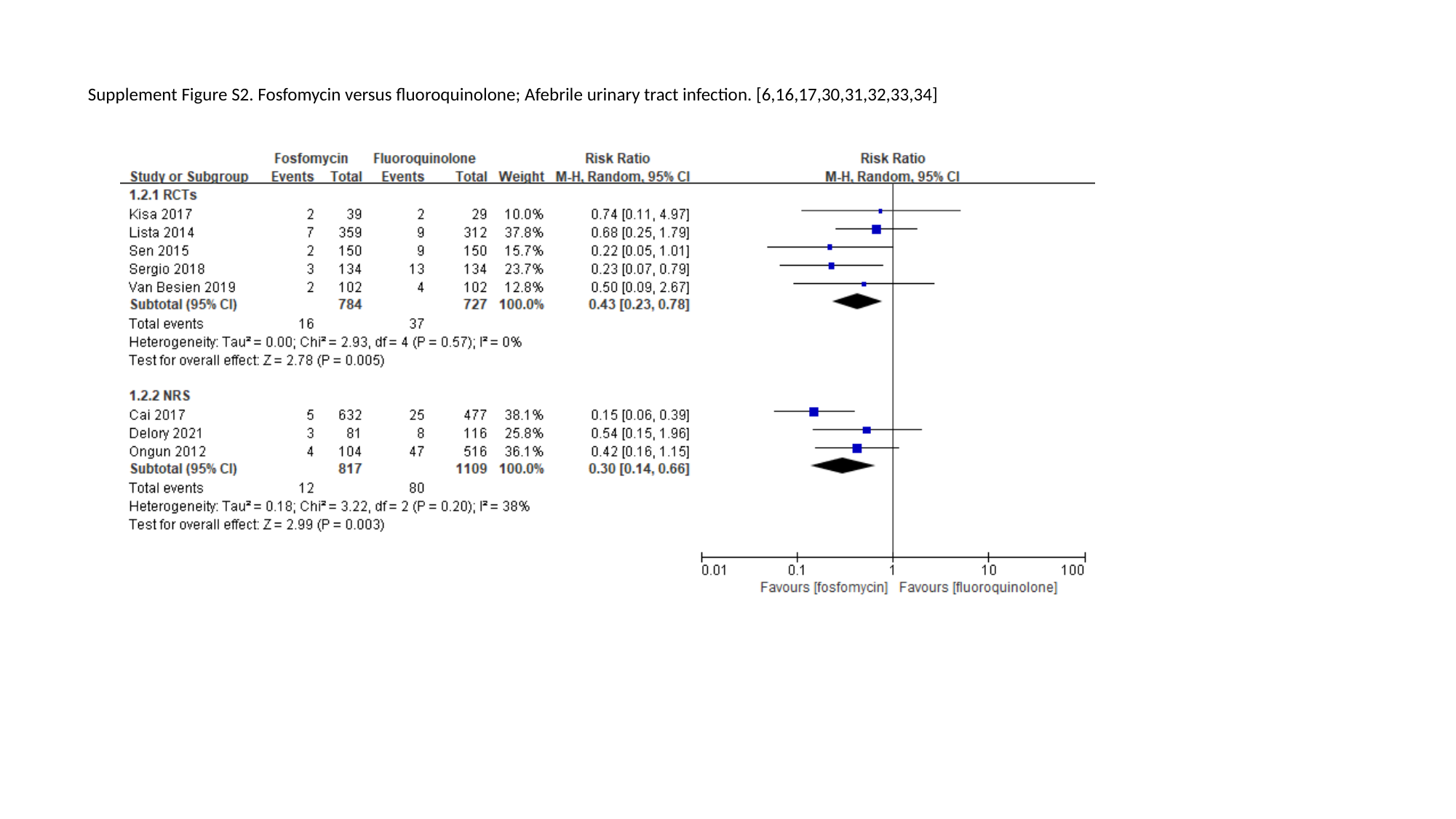

Supplement Figure S2. Fosfomycin versus fluoroquinolone; Afebrile urinary tract infection. [6,16,17,30,31,32,33,34]

## Slide 3
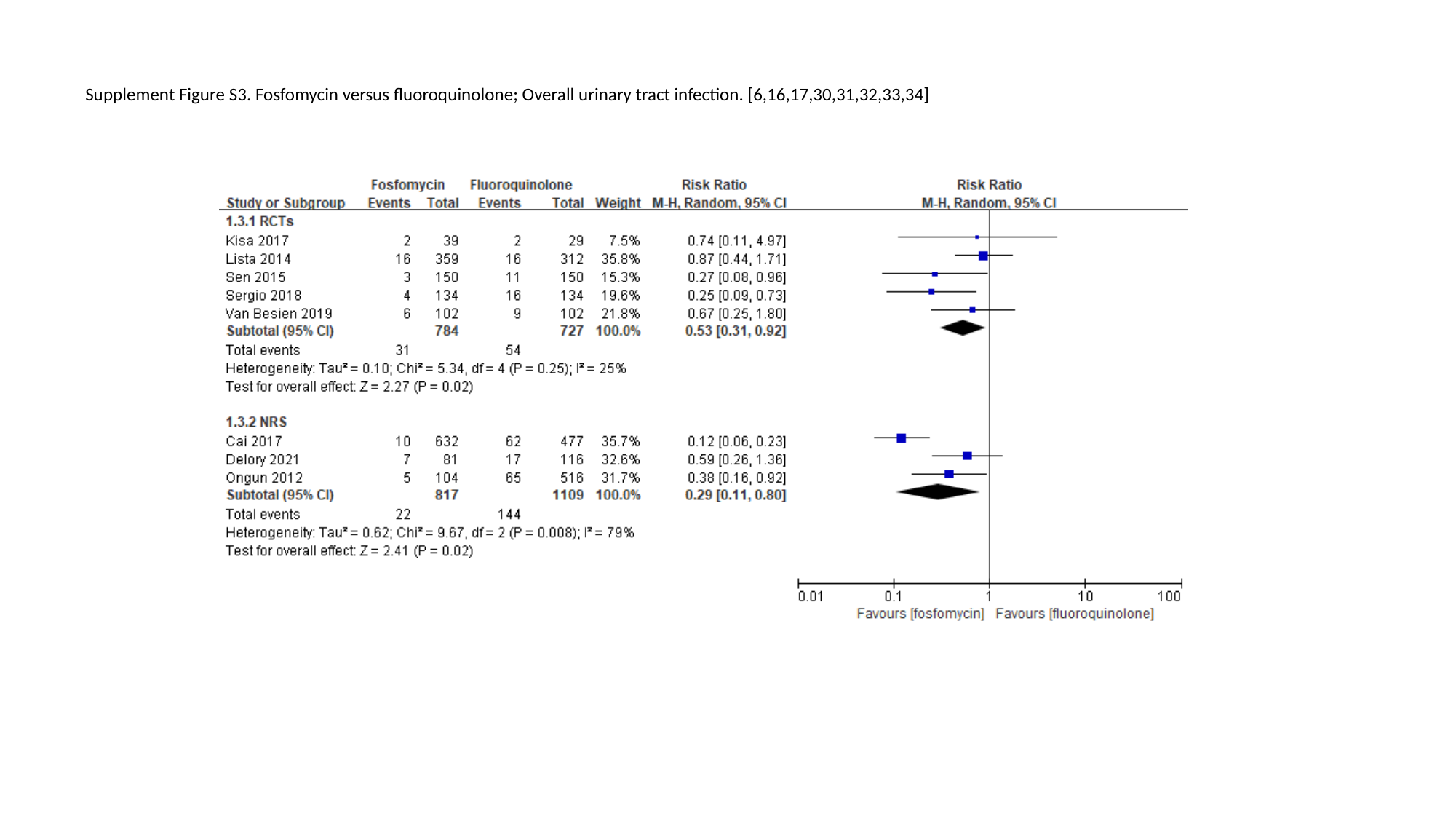

Supplement Figure S3. Fosfomycin versus fluoroquinolone; Overall urinary tract infection. [6,16,17,30,31,32,33,34]

## Slide 4
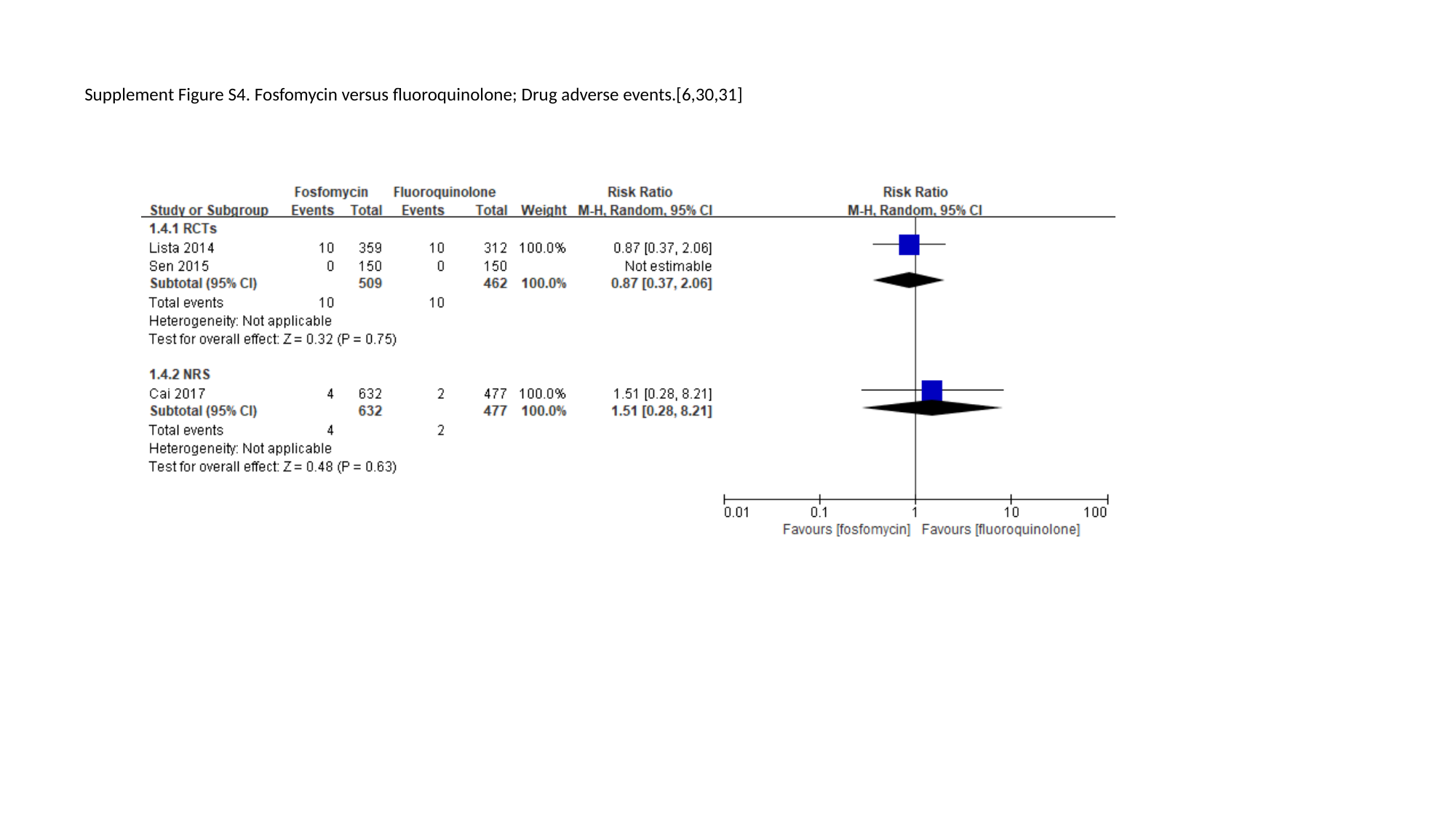

Supplement Figure S4. Fosfomycin versus fluoroquinolone; Drug adverse events.[6,30,31]

## Slide 5
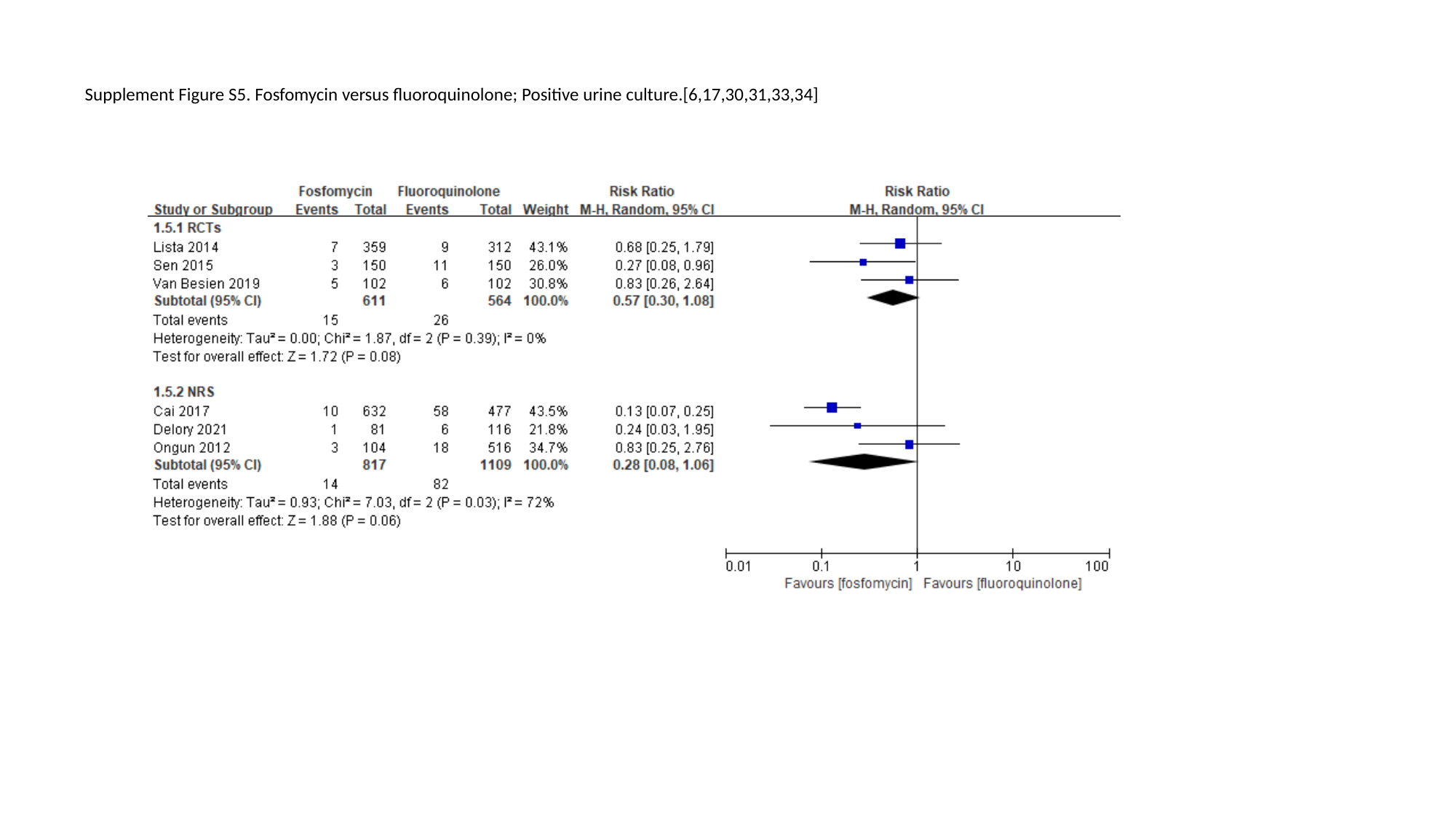

Supplement Figure S5. Fosfomycin versus fluoroquinolone; Positive urine culture.[6,17,30,31,33,34]

## Slide 6
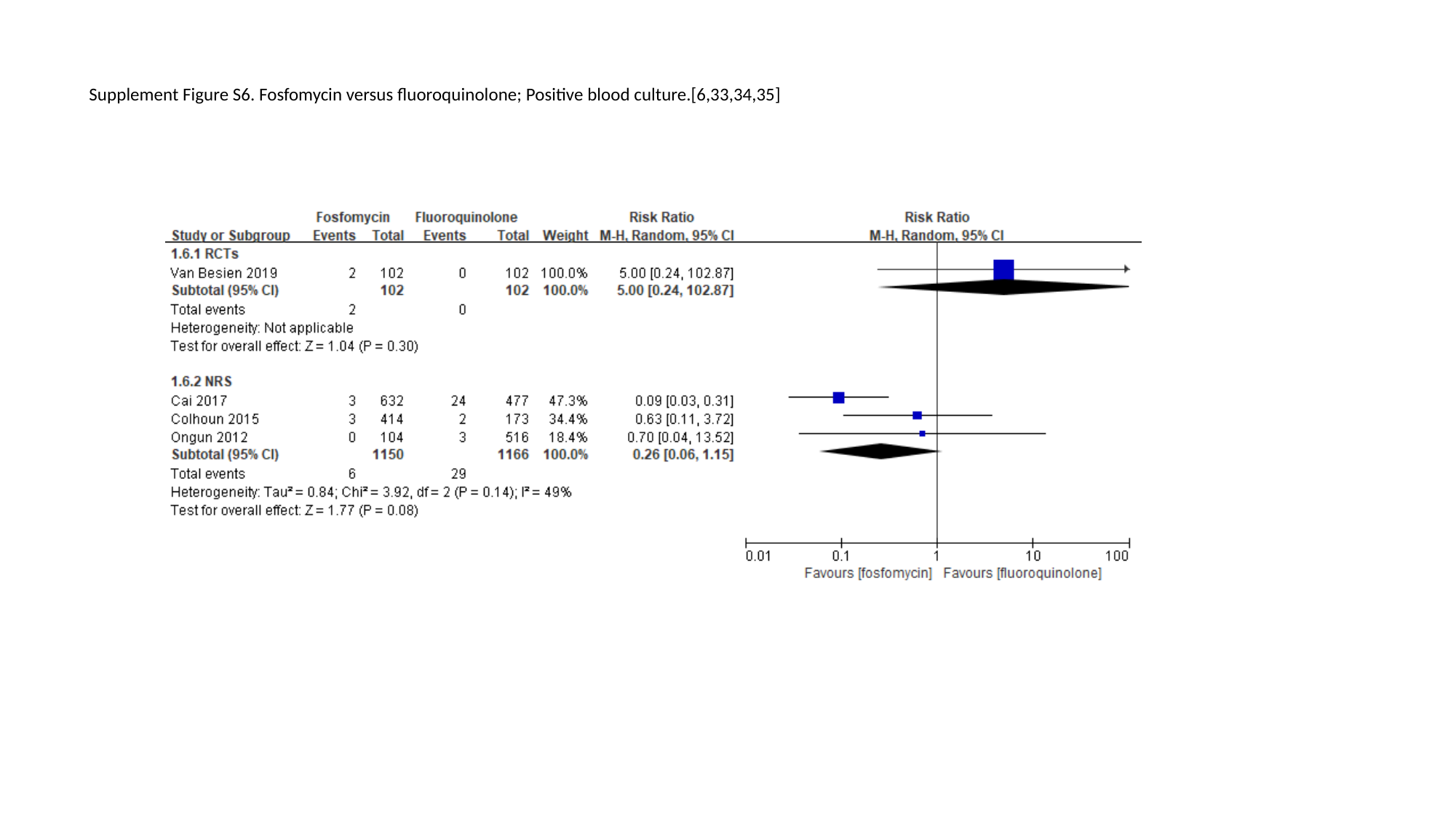

Supplement Figure S6. Fosfomycin versus fluoroquinolone; Positive blood culture.[6,33,34,35]

## Slide 7
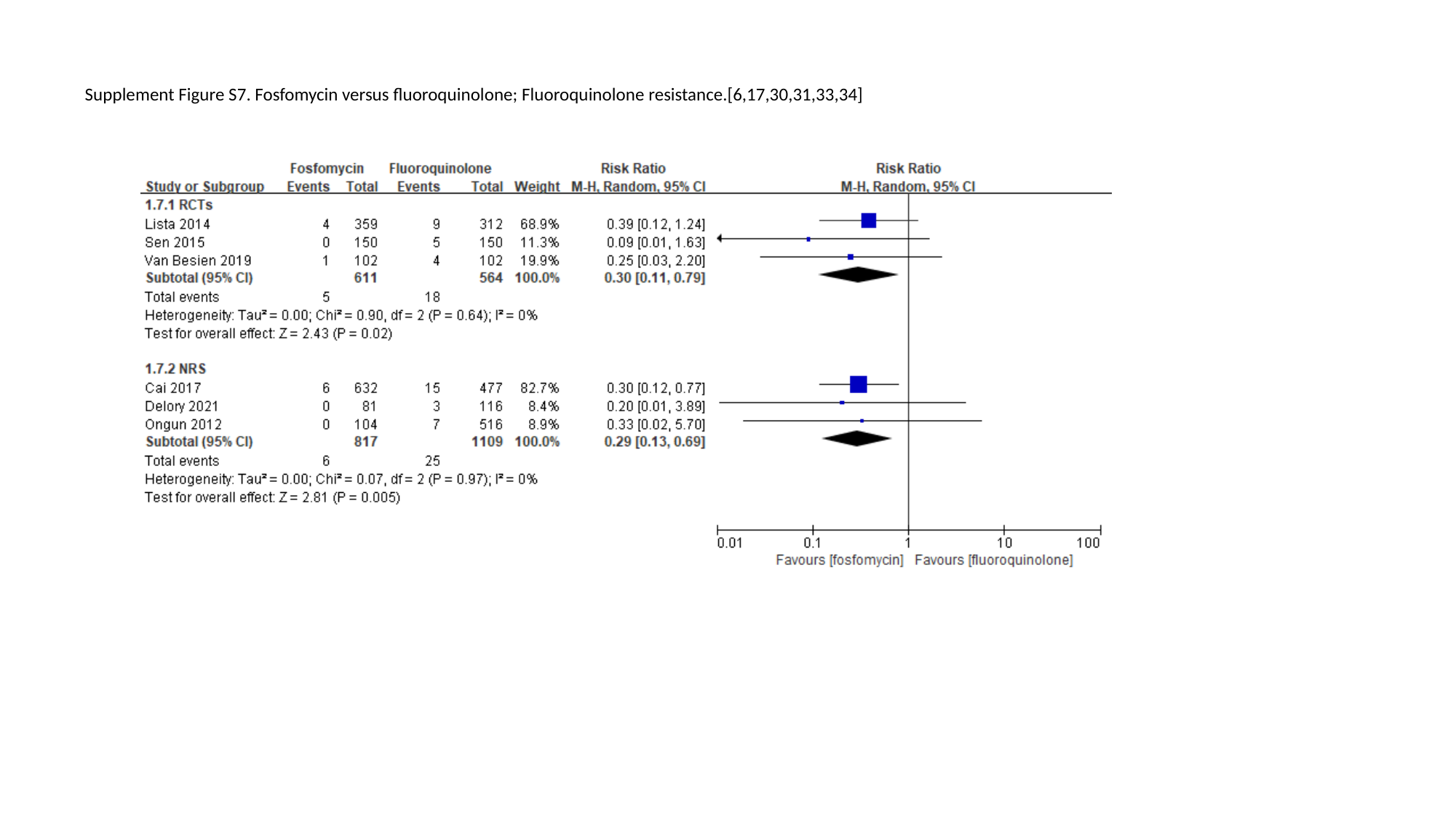

Supplement Figure S7. Fosfomycin versus fluoroquinolone; Fluoroquinolone resistance.[6,17,30,31,33,34]

## Slide 8
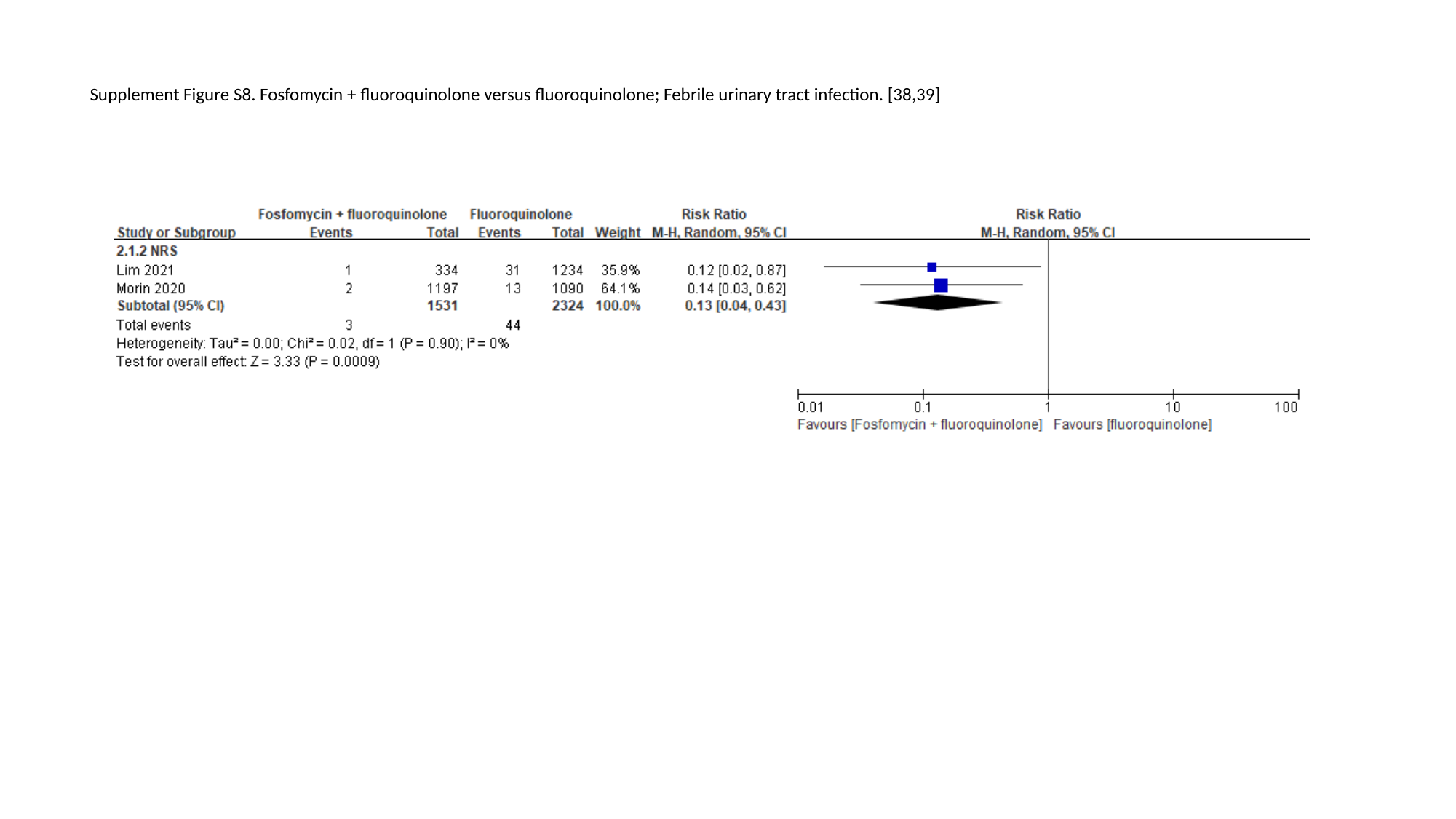

Supplement Figure S8. Fosfomycin + fluoroquinolone versus fluoroquinolone; Febrile urinary tract infection. [38,39]

## Slide 9
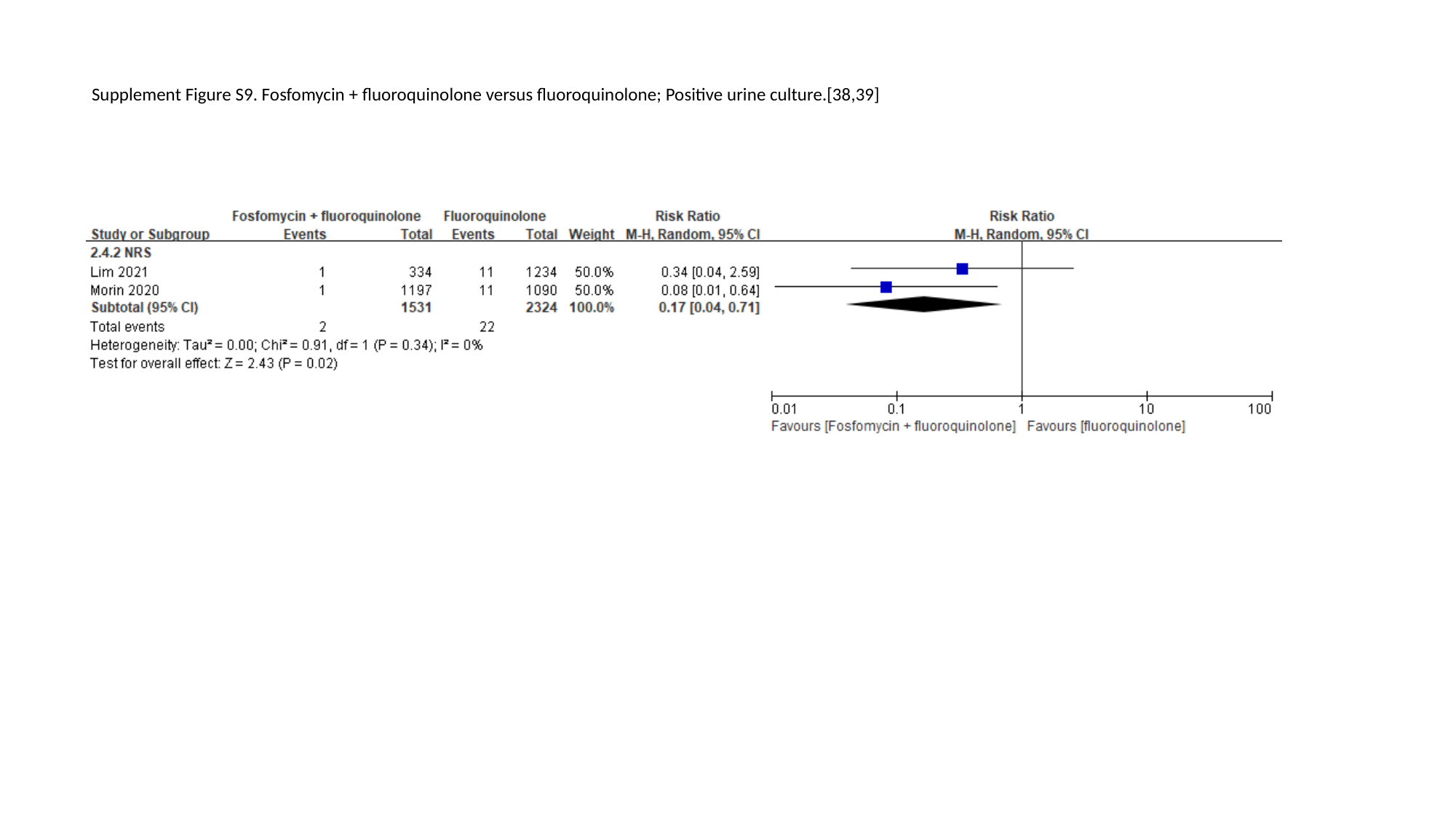

Supplement Figure S9. Fosfomycin + fluoroquinolone versus fluoroquinolone; Positive urine culture.[38,39]

## Slide 10
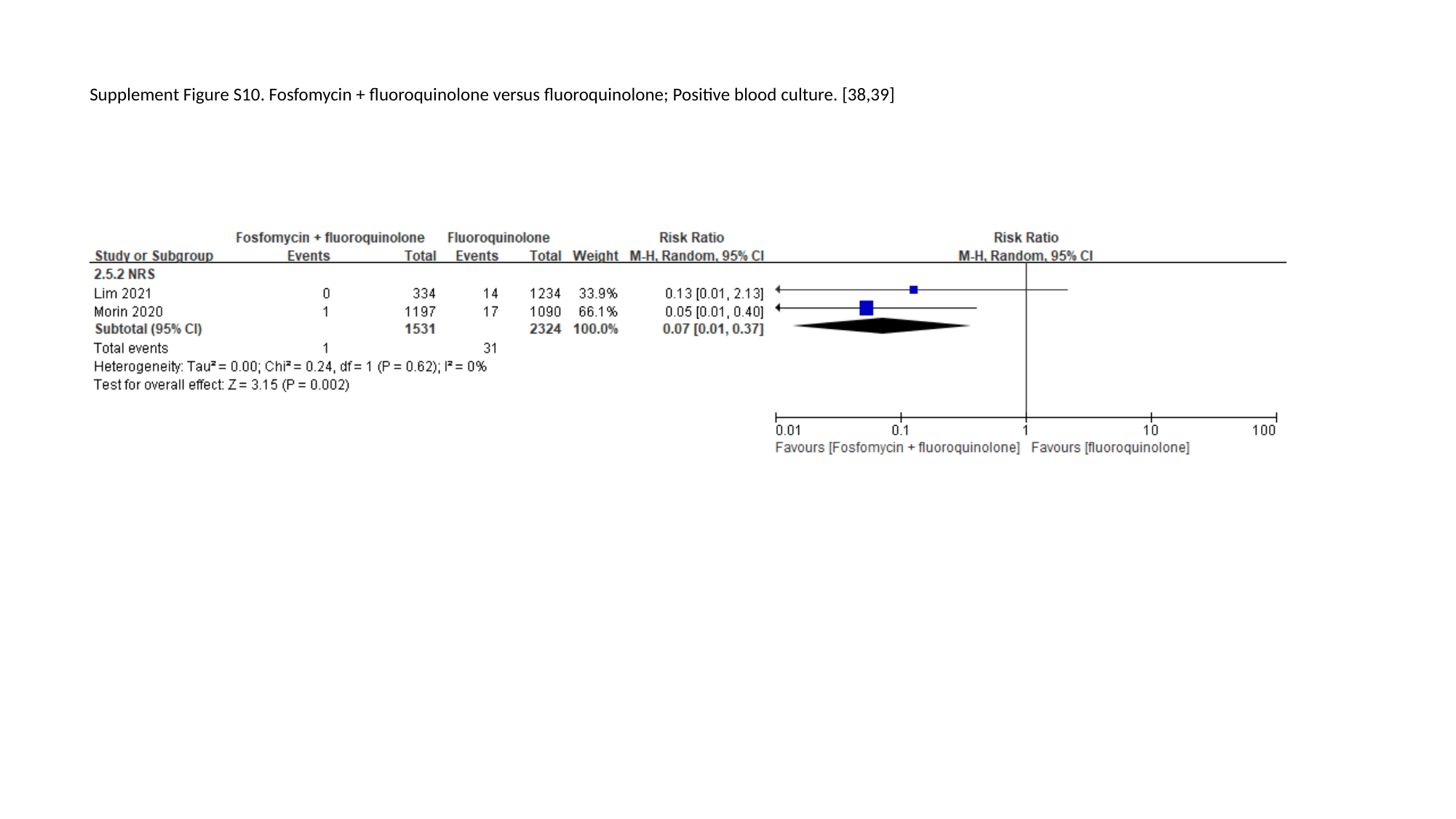

Supplement Figure S10. Fosfomycin + fluoroquinolone versus fluoroquinolone; Positive blood culture. [38,39]

## Slide 11
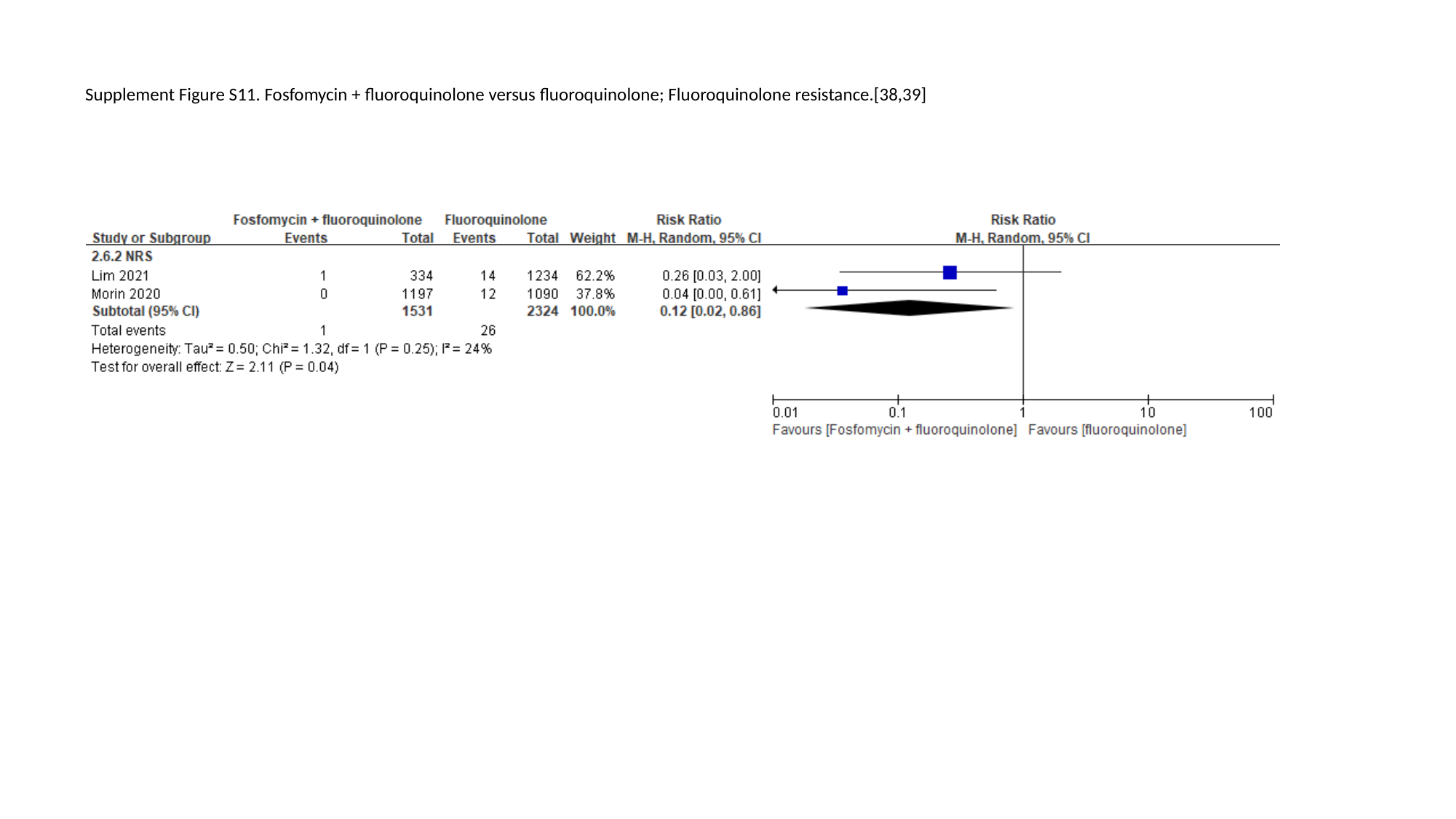

Supplement Figure S11. Fosfomycin + fluoroquinolone versus fluoroquinolone; Fluoroquinolone resistance.[38,39]

## Slide 12
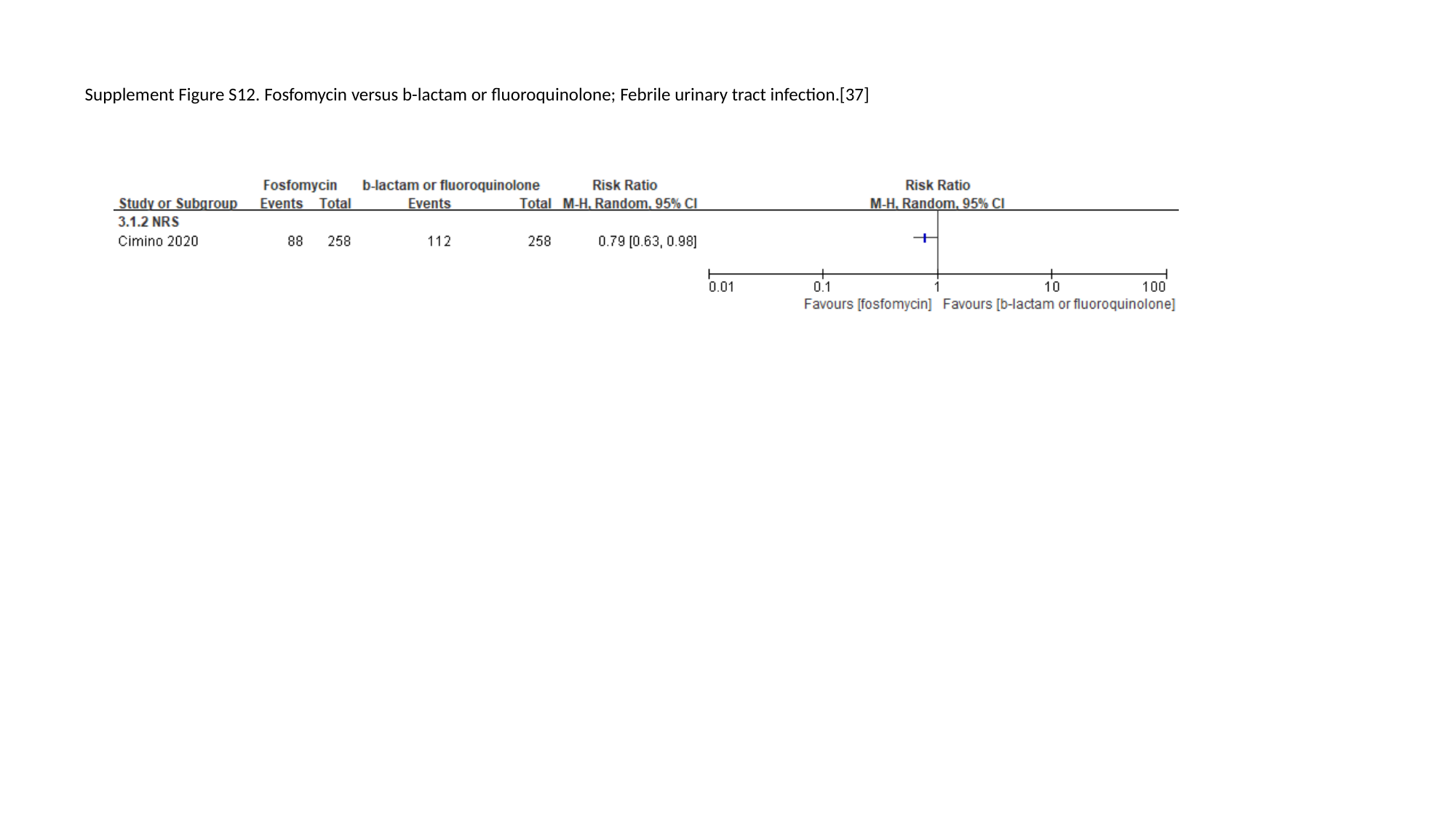

Supplement Figure S12. Fosfomycin versus b-lactam or fluoroquinolone; Febrile urinary tract infection.[37]

## Slide 13
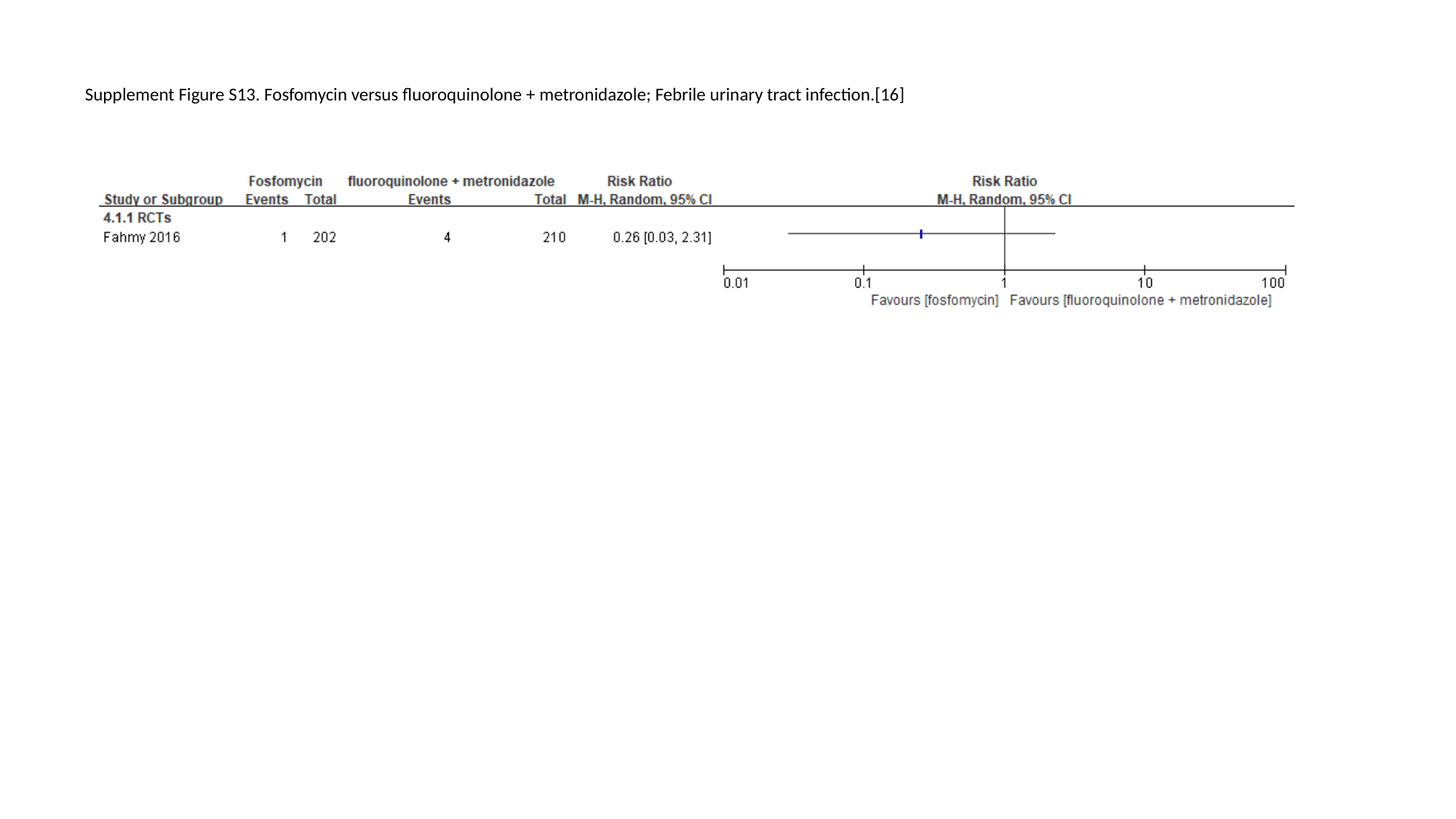

Supplement Figure S13. Fosfomycin versus fluoroquinolone + metronidazole; Febrile urinary tract infection.[16]

## Slide 14
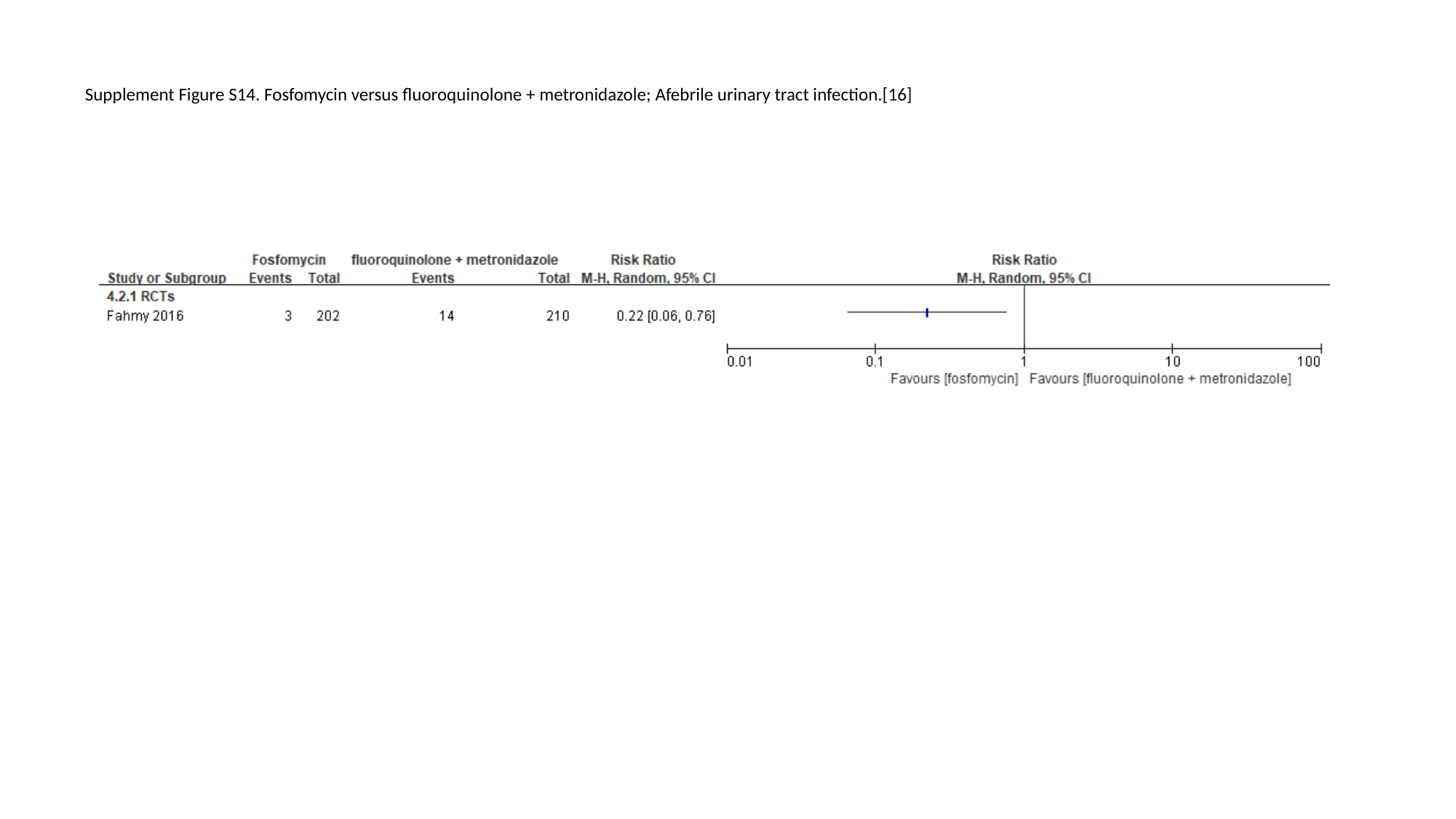

Supplement Figure S14. Fosfomycin versus fluoroquinolone + metronidazole; Afebrile urinary tract infection.[16]

## Slide 15
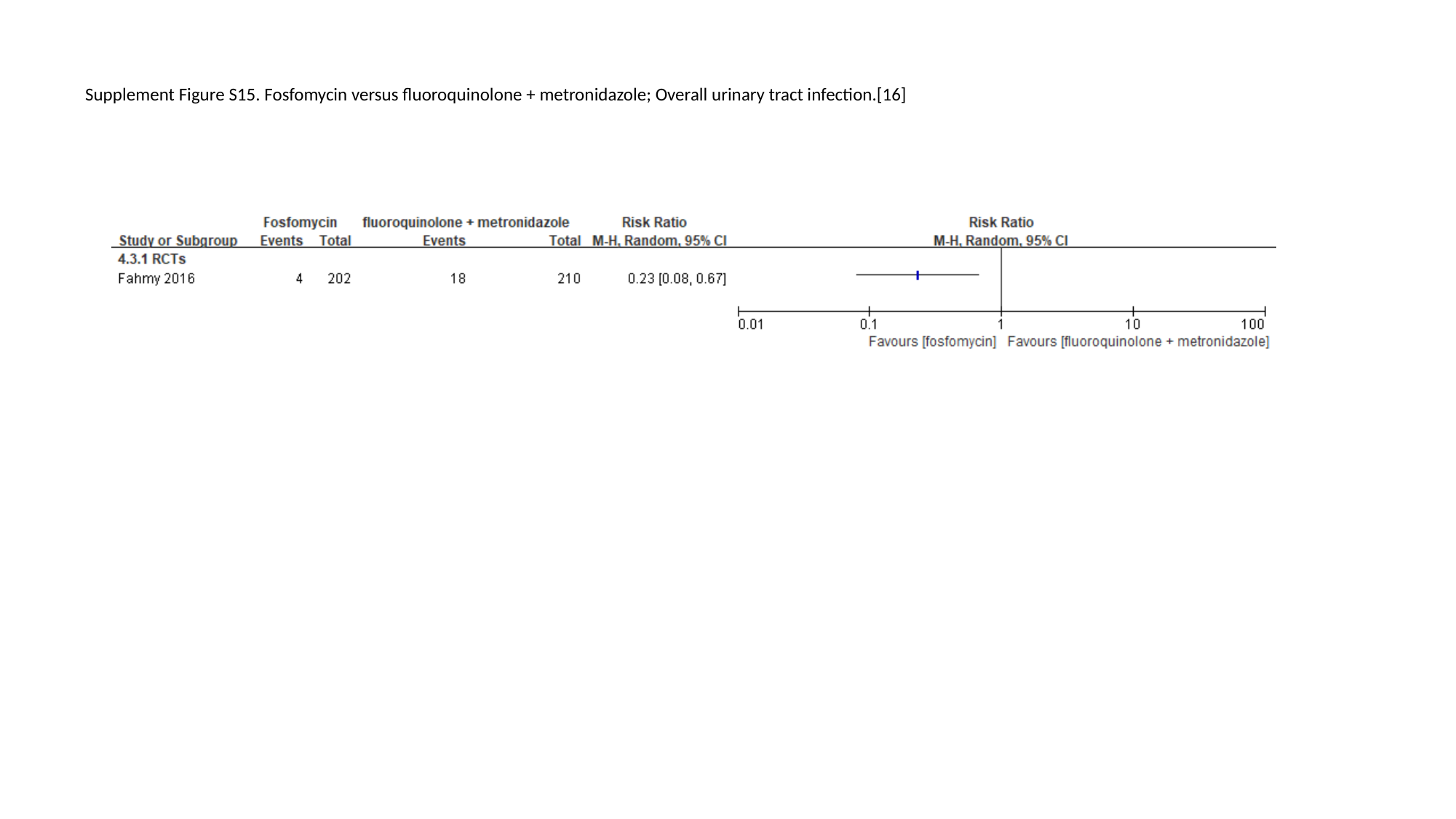

Supplement Figure S15. Fosfomycin versus fluoroquinolone + metronidazole; Overall urinary tract infection.[16]

## Slide 16
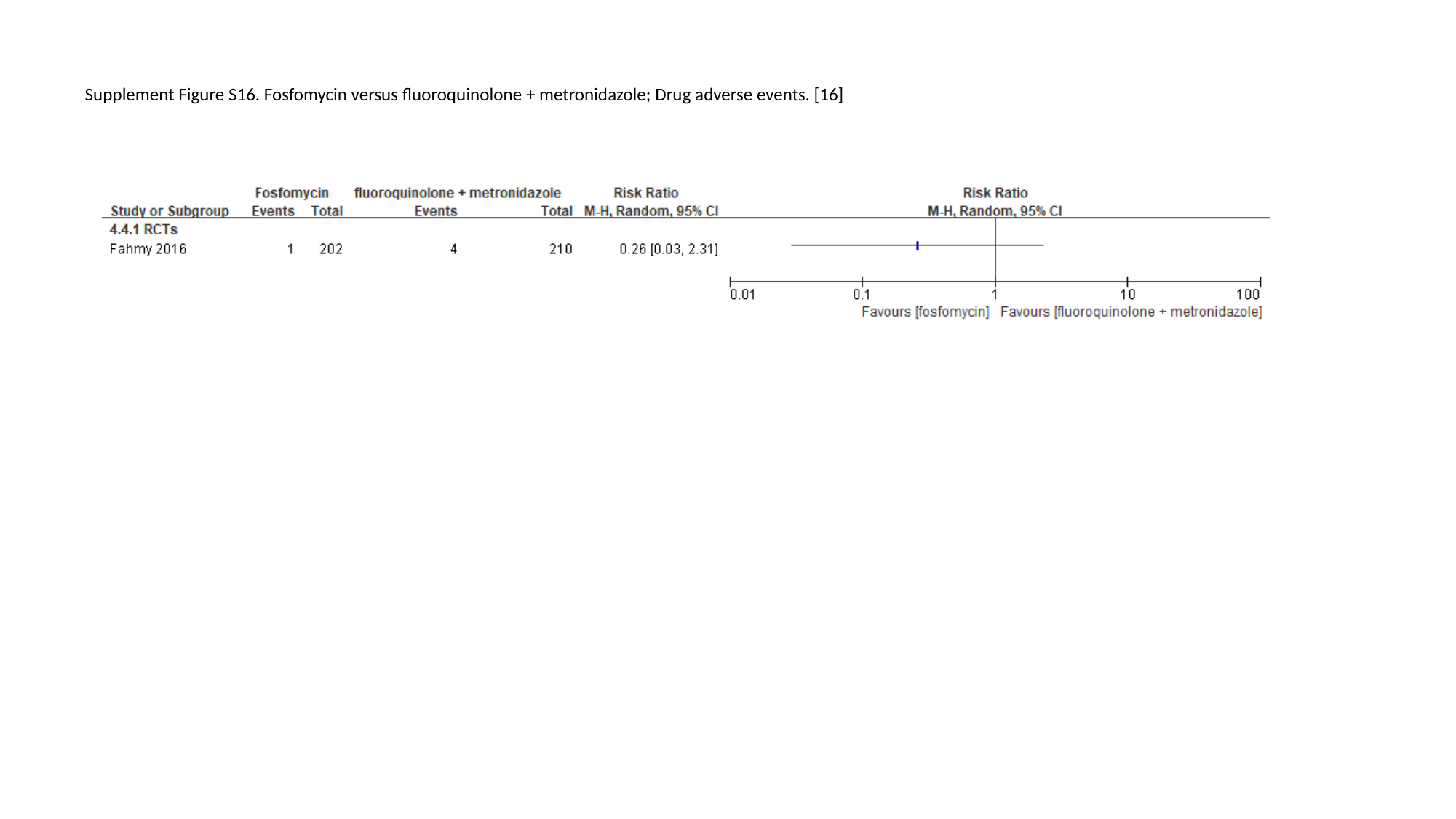

Supplement Figure S16. Fosfomycin versus fluoroquinolone + metronidazole; Drug adverse events. [16]

## Slide 17
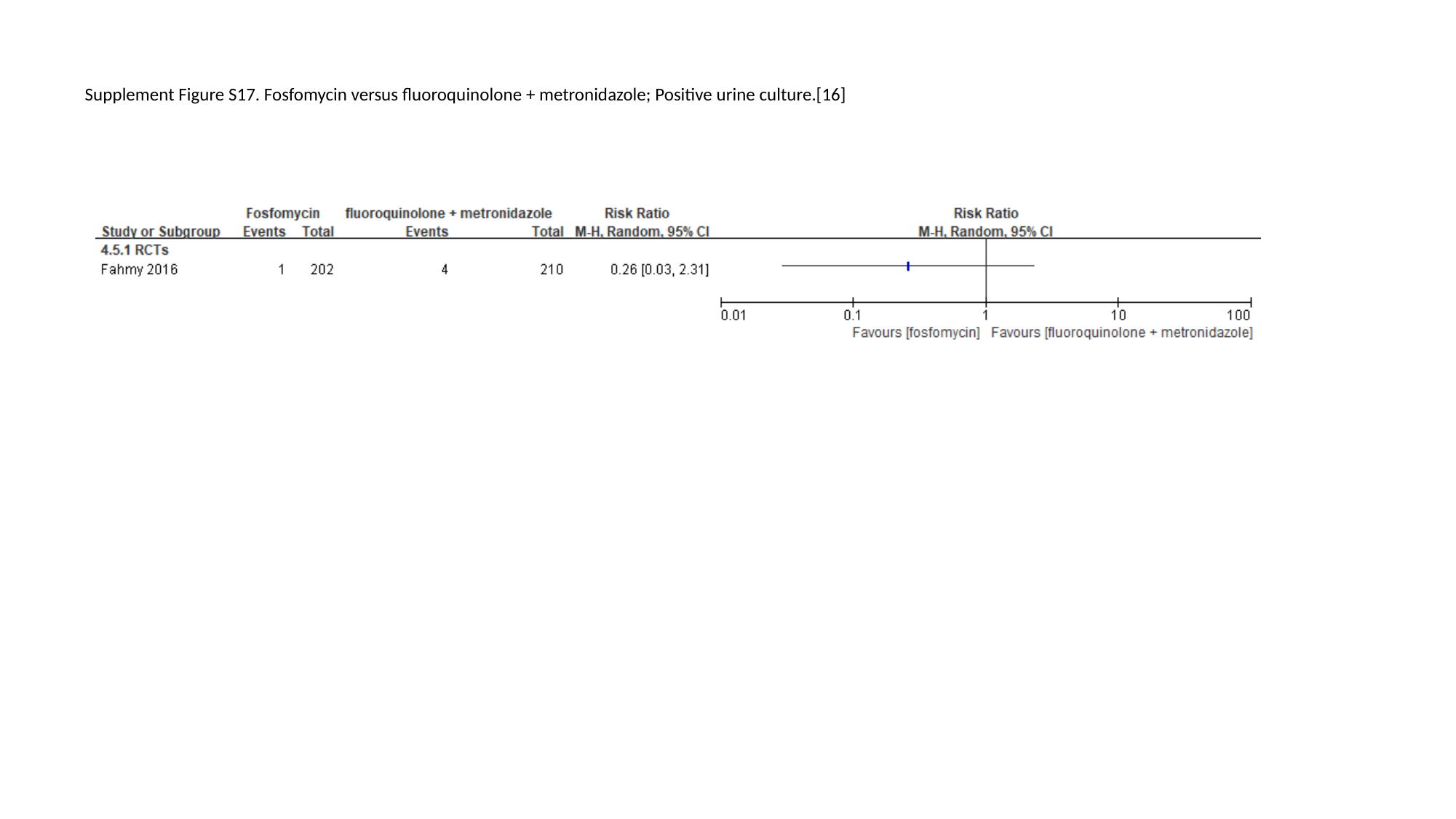

Supplement Figure S17. Fosfomycin versus fluoroquinolone + metronidazole; Positive urine culture.[16]

## Slide 18
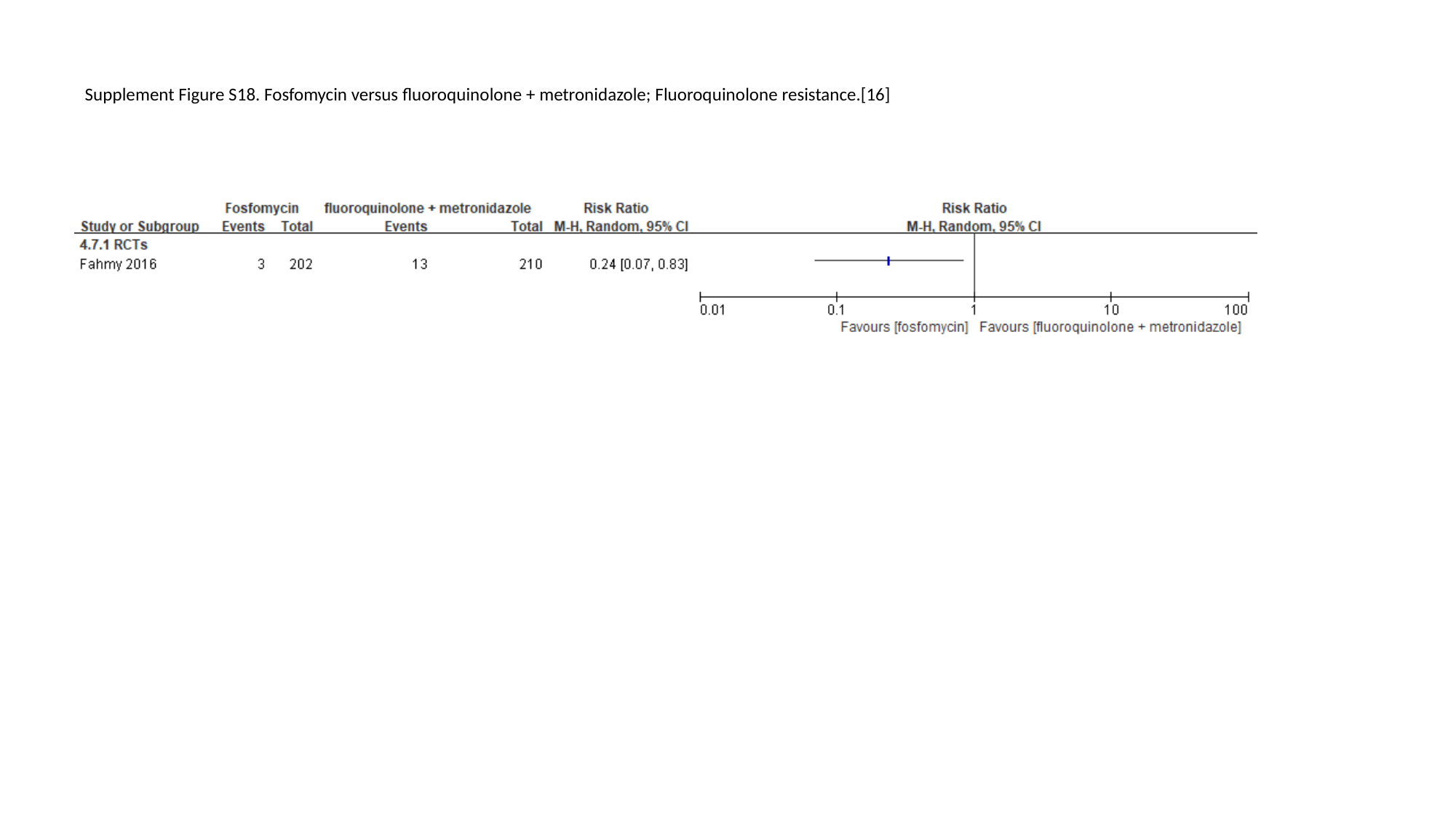

Supplement Figure S18. Fosfomycin versus fluoroquinolone + metronidazole; Fluoroquinolone resistance.[16]

## Slide 19
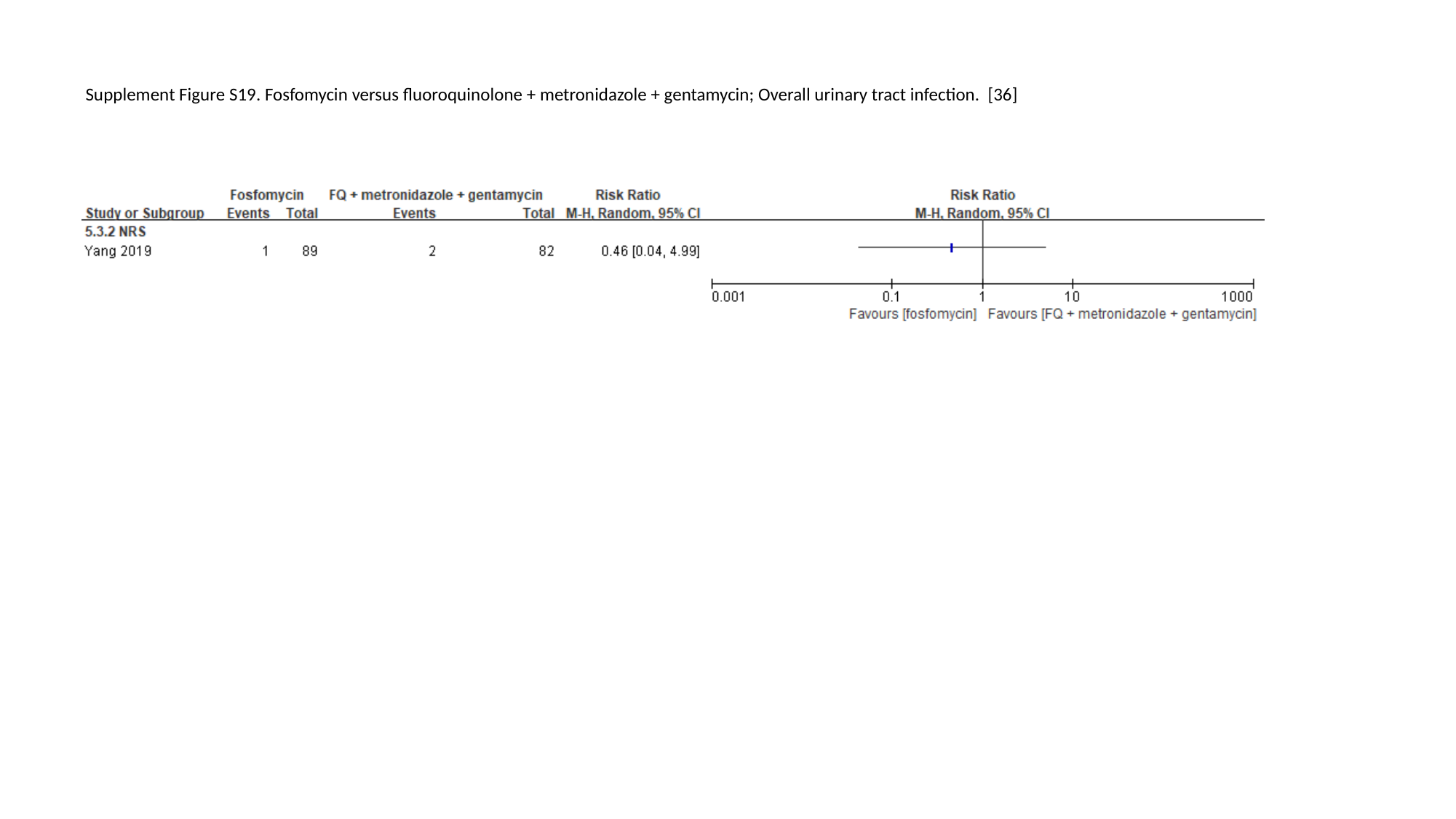

Supplement Figure S19. Fosfomycin versus fluoroquinolone + metronidazole + gentamycin; Overall urinary tract infection. [36]
