# Supplementary material for: Fosfomycin for Antibiotic Prophylaxis in Men Undergoing a Transrectal Prostate Biopsy: A Systematic Review and Meta-Analysis
Source: Medicina (Kaunas). 2023 May 10;59(5):911. doi: 10.3390/medicina59050911 (PMC10221023; doi:10.3390/medicina59050911)
Supplement: Supplementary file 1 [file medicina-59-00911-s001.zip › Supplement table and search strategy.pdf]

Characteristics of studies

Characteristics of included studies

Lim 2021

**Methods**	Study design: Retrospective cohort study<br>Study dates: January 2011 - June 2019<br>Setting: Single institution<br>Country: Korea<br>Study endpoint: 30 days
**Participants**	Inclusion criteria:<br>• men undergoing TRUS prostate biopsy<br>Exclusion criteria:<br>• FQ resistance treated with targeted antibiotics such as amikacin, tazobactam, as determined by the rectal swab culture<br>Screened: Not reported ; Eligible: 1578<br>Age (mean in years ±SD): FQ and FM 70 (SD: 8.1); FQ 70 (SD: 8.1)<br>PSA (ng/mL ±SD): FQ and FM 6.8 (SD: 8.5); FQ 7 (SD: 7.6)<br>Prostate volume (cc ±SD): FQ and FM 36.1 (SD: 19.9); FQ 32.8 (SD: 9.6)<br>DM: not available<br>Prostate cancer: FQ and FM 174; FQ 539<br>BPH: FQ and FM 169; FQ 664
**Interventions**	Intervention (n = 334): FQ and FM; ciprofloxacin for 1-2 days after the day of the biopsy (400 mg, intravenous [IV], twice daily)+ a single 3 g oral dose of FM the night before the procedure<br>Control (n = 1234): FQ; ciprofloxacin for 1-2 days after the day of the biopsy (400 mg, intravenous [IV], twice daily)
**Outcomes**	Primary outcomes:<br>Overall infectious complications within 1 month of TRUS prostate biopsy<br>• Fever, acute prostatitis, bacteremia, SIRS, and sepsis<br>Secondary outcomes:<br>Risk factors of infectious complications after TRUS prostate biopsy<br>• Antibiotic (FQ) exposure within 6 months<br>• Addition of FM
**Funding sources**	This study was supported by a grant (HCRI 20049) from the Chonnam National University Hwasun Hospital Institute for Biomedical Science.
**Declarations of interest**	None
**Notes**	Language of publication : English

Cai 2017

**Methods**	Study design: Retrospective cohort study<br>Study dates: January 2015 – September 2015<br>Setting: Multicenter<br>Country: Italy<br>Study endpoint: 1 month after procedure
**Participants**	Inclusion criteria:<br>• older than 18 years and candidates for prostate biopsy<br>• had a urine culture at the follow-up visit<br>Exclusion criteria:<br>• significant comorbidities (Charlson comorbidity index >3)<br>• anatomical abnormalities of the urinary tract<br>• previous symptomatic UTIs due to fluoroquinolone-resistant and fosfomycin-resistant strains<br>• positive urine culture at the procedure time<br>Screened: 1371 ; Eligible: 1109

	Age (mean in years ±SD): FM 65.9 (SD: 8.3); CIP 66.9 (SD: 8.9)<br>PSA (ng/mL ±SD): FM 7.14 (SD: 4.31); CIP 7.69 (SD: 5.09)<br>Prostate volume (cc ±SD): FM 36.1 (SD: 19.9); CIP 32.8 (SD: 9.6)<br>DM: FM 62; CIP 40<br>Prostate cancer: FM 285; CIP 210<br>BPH: FM 332; CIP 251
**Interventions**	Intervention (n = 632): FM; 3 g FM orally 3 h before and 3 g 24 h after the first administration<br>Control (n = 477): CIP; 500 mg CIP as prophylaxis administered orally twice daily for 5 days starting 1 day before the procedure
**Outcomes**	Primary outcomes:<br>Rate of symptomatic UTIs<br>• body temperature <38 °C and dysuria accompanied by pyuria<br>• Fever ≥38 °C accompanied by one symptom of the lower urinary tract (i.e., urgency, frequency, dysuria, or suprapubic tenderness)<br>• Fever ≥38 °C, accompanied by chills and malaise<br>• urosepsis as defined in the EAU guidelines on urological infections<br>Secondary outcomes:<br>Adverse drug reactions
**Funding sources**	None
**Declarations of interest**	Tommaso Cai has received grant from Zambon as speaker at scientific meeting; Kurt Naber and Florian Wagenlehner are consultant for and have received research support and grant as speakers at scientific meeting from Zambon.
**Notes**	Language of publication : English

Sergio 2018

**Methods**	Study design: Randomized controlled trial<br>Study dates: May 2016 – September 2017<br>Setting: Single center<br>Country: Philippines<br>Study endpoint: Not reported
**Participants**	Inclusion criteria:<br>• underwent prostate biopsy<br>Screened: Not reported ; Eligible: 268<br>Age (mean in years ±SD): not available<br>PSA (ng/mL ±SD): not available<br>Prostate volume (cc ±SD): not available<br>DM: not available<br>Prostate cancer: not available<br>BPH: not available
**Interventions**	Intervention (n = 134): FM; 3 gram sachet dissolved in 1/2 glass water 60 mins prior to biopsy<br>Control (n = 134): CIP; 500 mg twice daily capsules administered for five days starting one day before the biopsy
**Outcomes**	Primary outcomes:<br>Post-procedural febrile and afebrile infectious complications<br>Secondary outcomes: not available
**Funding sources**	Not reported
**Declarations of interest**	Not reported
**Notes**	Language of publication : English

Cimino 2020

**Methods**	Study design: Randomized controlled trial<br>Study dates: September 2016 – March 2017<br>Setting: Multicenter<br>Country: Italy<br>Study endpoint: 4 wk after biopsy
**Participants**	Inclusion criteria:<br>• underwent prostate biopsy<br>Exclusion criteria:<br>• not provide enough data; age, PSA, and Charlson comorbidity index<br>• lost at follow-up<br>• UTIs prior to the procedure; dipstick analysis<br>Screened: 728 ; Eligible: 516<br>Age (mean in years ±SD): FM 66 (SD: 6.7); ß-lactame or FQ 67 (SD: 8.1)<br>PSA (ng/mL ±SD): FM 7.59 (SD: 4.7); ß-lactame or FQ 8.0 (SD: 5.1)<br>Prostate volume (cc ±SD): not available<br>DM: not available<br>Prostate cancer: not available<br>BPH: not available
**Interventions**	Intervention (n = 258): FM; a single dose of 3 g oral FM the night before the procedure and 12 h after the procedure<br>Control (n = 258): ß-lactame or FQ; a single dose 2 before the procedure
**Outcomes**	Primary outcomes:<br>major complications (ClavienDindo ≥2)<br>Secondary outcomes: not available
**Funding sources**	None
**Declarations of interest**	None
**Notes**	Language of publication : English

Colhoun 2015

**Methods**	Study design: Retrospective cohort study<br>Study dates: November 2012 – Octer 2014<br>Setting: Single center<br>Country: America<br>Study endpoint: within 14 days of biopsy
**Participants**	Inclusion criteria:<br>• underwent prostate biopsy<br>Exclusion criteria: not available<br>Screened: 621 ; Eligible: 587<br>Age (mean in years ±SD): not available<br>PSA (ng/mL ±SD): not available<br>Prostate volume (cc ±SD): not available<br>DM: not available<br>Prostate cancer: not available<br>BPH: not available
**Interventions**	Intervention (n = 414): FM; fosfomycin 3g PO 2-3 hours before TRUS prostate biopsy<br>Control (n = 173): CIP; standard prophylaxis with 6 doses of ciprofloxacin
**Outcomes**	Primary outcomes:<br>signs of infectious complications within 14 days of biopsy and corresponding bacterial culture results

	Secondary outcomes: not available
**Funding sources**	None
**Declarations of interest**	Not reported
**Notes**	Language of publication : English

Delory 2021

**Methods**	Study design: Randomized controlled trial<br>Study dates: April 2017 – June 2019<br>Setting: Single center<br>Country: French<br>Study endpoint: 30 days after biopsy
**Participants**	Inclusion criteria:<br>Men undergoing TRUS prostate biopsy for suspicion of prostate cancer<br>Exclusion criteria:<br>• fever on the day of TRUS prostate biopsy (≥ 38.0 C)<br>• allergy or intolerance to FQ and/or FM<br>• positive urine culture requiring antibiotic therapy in the week before TRUS prostate biopsy<br>Screened: 222 ; Eligible: 197<br>Age (mean in years ±SD): FM 67.8 (SD: 6.9); FQ 67.4 (SD: 8.1)<br>PSA (ng/mL ±SD): not available<br>Prostate volume (cc ±SD): FM 50 (SD: 18.5); FQ 44 (SD: 18.5)<br>DM: not available<br>Prostate cancer: FM 19; FQ 95<br>BPH: not availble
**Interventions**	Intervention (n = 81): FM; a single dose of oral FM 3g after fasting, 2 h prior to the biopsies<br>Control (n = 116): FQ; a single dose of ciprofloxacin (500 mg), or levofloxacin (500 mg), or ofloxacin (400 mg) after fasting, 2 hr prior to the biopsies
**Outcomes**	Primary outcomes:<br>self-reported TRUS prostate biopsy UTI<br>• at least one of the following signs or symptoms:<br>• pelvic pain, pain/burning when urinating, frequent urination, urgency, leaking, acute urinary retention, hematuria associated or not with fever ≥ 38 °C or chills<br>Secondary outcomes:<br>• microbiologically documented post-TRUSPB UTI based on urine<br>• self-reported antibiotic intake<br>• hospitalization (all causes and related to post TRUS-PB UTI)<br>• post TRUS-PB self-reported adverse events (digestive, neurological, cutaneous, musculoskeletal, and urinary symptoms)
**Funding sources**	None
**Declarations of interest**	None
**Notes**	Language of publication : English

VanBesien 2019

**Methods**	Study design: Randomized controlled trial<br>Study dates: December 2015 – December 2017<br>Setting: Multicenter<br>Country: Belgium

	Study endpoint: 1 month after biopsy
**Participants**	Inclusion criteria:<br>• undergoing a prostate biopsy<br>Exclusion criteria:<br>• cancelled prostate biopsy<br>Screened: 209 ; Eligible: 204<br>Age (mean in years ±SD): FM 64 (SD: 5.1); FQ 67 (SD: 4.7)<br>PSA (ng/mL ±SD): FM 7.1 (SD: 6.6); FQ 7.2 (SD: 6.0)<br>PSA (ng/mL ±SD): not available<br>Prostate volume (cc ±SD): not available<br>DM: FM 12; FQ 8<br>Prostate cancer: FM 83; FQ 73<br>BPH: not available
**Interventions**	Intervention (n = 102): FM; fosfomycine 3 g 2 h before the biopsy<br>Control (n = 102): FQ; a tablet of ciprofloxacin 500 mg 2 h before the scheduled biopsy
**Outcomes**	Primary outcomes:<br>• minor afebrile infectious complications (epididymitis or cystitis)<br>• major febrile infectious complications (prostatitis, pyelonephritis, urosepsis)<br>Secondary outcomes:<br>• risk factors for FQ resistance<br>• risk factors for infectious complications
**Funding sources**	None
**Declarations of interest**	None
**Notes**	Language of publication : English

Sen 2015

**Methods**	Study design: Randomized controlled trial<br>Study dates: May 2014 – Feb 2015<br>Setting: Single center<br>Country: Turkey<br>Study endpoint: 1 month after biopsy
**Participants**	Inclusion criteria:<br>• undergoing a prostate biopsy<br>Exclusion criteria:<br>• history of UTI, indwelling urinary catheters, and antibiotic use within a month of study initiation<br>Screened: 300 ; Eligible: 300<br>Age (mean in years ±SD): FM 63.5 (SD: 7.5); FQ 62.9 (SD: 7.6)<br>PSA (ng/mL ±SD): FM 12.9 (SD: 1.8); FQ 12.0 (SD: 1.2)<br>DM: not available<br>Prostate volume (cc ±SD): FM 53.1 (SD: 22.5); FQ 51.3 (SD: 24.6)<br>Prostate cancer: FM 36; FQ 39<br>BPH: FM 75; FQ 55
**Interventions**	Intervention (n = 102): FM; a single dose of 3 g oral fosfomycin the night before the procedure<br>Control (n = 102): FQ; 500 mg oral ciprofloxacin 60min before the procedure
**Outcomes**	Primary outcomes:<br>Post-procedural febrile and afebrile infectious complication and pathological characteristics

	• Afebrile UTI: fever < 38°C and dysuria accompanied by pyuria<br>• Pyuria: presence of > 10 white blood cells in 1 mm3 of midstream urine<br>• Febrile UTI: fever 38°C accompanied by one symptom of the lower urinary tract (i.e., urgency, frequency, dysuria, or suprapubic tenderness), with or without a positive urine culture<br>Secondary outcomes: not available
**Funding sources**	None
**Declarations of interest**	None
**Notes**	Language of publication : English

Fahmy 2016

**Methods**	Study design: Randomized controlled trial<br>Study dates: Feburary 2012 – June 2015<br>Setting: Single center<br>Country: Egypt<br>Study endpoint: within 4 weeks of TRUSBx
**Participants**	Inclusion criteria:<br>• undergoing TRUS prostate biopsy<br>Exclusion criteria:<br>• history of allergy or intolerance to anyone of the study drugs<br>• UTI with positive urine culture<br>• indwelling urinary catheters<br>• antibiotic use during the previous 4 weeks<br>Screened: 440 ; Eligible: 412<br>Age (mean in years ±SD): FM 68.8 (SD: 4.2); Standard FQ 62.5 (SD: 2.8)<br>PSA (ng/mL ±SD): FM 23.9 (SD: 5.8); Standard FQ 17.8 (SD: 3.2)<br>Prostate volume (cc ±SD): FM 67.3 (SD: 31.2); Standard FQ 59.8 (SD: 28.5)<br>DM: not available<br>Prostate cancer: not available<br>BPH: not available
**Interventions**	Intervention (n = 202): FM; single-dose fosfomycin (3 g, orally) 1–2 h before TRUS prostate biopsy<br>Control (n = 210): FQ; oral ciprofloxacin 500 mg and metronidazole 500 mg at least 1 h before biopsy and continued this twice daily for 3 days before biopsy
**Outcomes**	Primary outcomes:<br>occurrence of post biopsy infectious complications<br>• fever (≥ 38 °C)<br>• rigours and/or any one of the following lower urinary symptoms e.g. dysuria, frequency, urgency or suprapubic pain<br>Secondary outcomes: not available
**Funding sources**	None
**Declarations of interest**	None
**Notes**	Language of publication : English

Kisa 2017

**Methods**	Study design: Randomized controlled trial<br>Study dates: May 2014 – October 2014<br>Setting: Single center<br>Country: Turkey

	Study endpoint: 4th weeks
**Participants**	Inclusion criteria:<br>• TRUS prostate biopsy was planned because of prostate cancer suspicion<br>Exclusion criteria:<br>• cotamination of pre-biopsy swabs<br>Screened: 155 ; Eligible: 68<br>Age (mean in years ±SD): not available<br>PSA (ng/mL ±SD): not available<br>Prostate volume (cc ±SD): not available<br>DM: not available<br>Prostate cancer: not available<br>BPH: not available
**Interventions**	Intervention (n = 39): FM;<br>• patients with no risk factors, using single dose fosfomycin the night before the biopsy, prophylaxis was started before getting the swab results<br>• those with risk factors, took fosfomycin according to the swab results<br>Control (n = 29): FQ;<br>• patients with no risk factors, using ciprofloxacin twice daily for 5 days, beginning the day before, prophylaxis was started before getting the swab results<br>• those with risk factors, took oral ciprofloxacin according to the swab results<br>Risk factors:<br>ciprofloxacin or other antibiotic use in the last 6 months, DM, urethral catheterization, genitourinary system operation history
**Outcomes**	Primary outcomes:<br>Infectious complication rates<br>• asymptomatic bacteriuria<br>• UTI without fever<br>• Fever<br>Secondary outcomes: not available
**Funding sources**	Not reported
**Declarations of interest**	None
**Notes**	Language of publication : English

Lista 2014

**Methods**	Study design: Randomized controlled trial<br>Study dates: September 2009 – December 2010<br>Setting: Single center<br>Country: Spain<br>Study endpoint: a week, a month and 3 months
**Participants**	Inclusion criteria:<br>• indication of a TRPB<br>Exclusion criteria:<br>• allergy or intolerance to any of the study drugs<br>Screened: 700 ; Eligible: 671<br>Age (mean in years ±SD): not available<br>PSA (ng/mL ±SD): not available<br>Prostate volume (cc ±SD): not available

	DM: not available<br>Prostate cancer: not available<br>BPH: not available
**Interventions**	Intervention (n = 359): FMT;<br>• received 3 g FM prophylaxis in 2 doses orally (24 h before and 24 h after the biopsy)<br>Control (n = 312): FQ;<br>• received ciprofloxacin 500 mg orally every 12 h for 5 days (total 10 tablets)
**Outcomes**	Primary outcomes: efficacy and safety<br>• Digestive intolerance<br>• severe allergic reaction<br>• fever, hemospermia, hematuria, rectal bleeding and urinary retention<br>Secondary outcomes: not available
**Funding sources**	None
**Declarations of interest**	None
**Notes**	Language of publication : Spanish

Morin 2020

**Methods**	Study design: Retrospective cohort study<br>Study dates: January 2012 – December 2015<br>Setting: Multi center<br>Country: Canada<br>Study endpoint: 1 month
**Participants**	Inclusion criteria:<br>• underwent a prostate biopsy<br>Exclusion criteria:<br>• did not receive either CIP or CIP/FM combination for antibioprophylaxis prior to the biopsy<br>• incomplete demographic data<br>Screened: 2287 ; Eligible: 2304<br>Age (mean in years ±SD): CIP+FM 65.0 (SD: 7.5); CIP 65.2 (SD: 7.7)<br>PSA (ng/mL): CIP+FM 6.0; CIP 5.7<br>Prostate volume (cc ±SD): not available<br>DM: CIP+FM 142; CIP 132<br>Prostate cancer: CIP+FM 773; CIP 626<br>BPH: CIP+FM 500; CIP 448
**Interventions**	Intervention (n = 1197): CIP+FM;<br>• CIP 500 mg and oral fosfomycin tromethamine 3 g two hours prior to the biopsy<br>Control (n = 1090): CIP;<br>• oral CIP 500 mg two hours prior to the biopsy
**Outcomes**	Primary outcomes: post biopsy urosepsis<br>• UTI with bacteremia<br>• UTI with SIRS<br>Secondary outcomes: not available
**Funding sources**	This research was made possible by an unrestricted educational grant from Sanofi.
**Declarations of interest**	Not available

**Notes**	Language of publication : English

Ongun 2012

**Methods**	Study design: Retrospective cohort study<br>Study dates: January 2010 – July 2011<br>Setting: Single center<br>Country: Turkey<br>Study endpoint: 1 month
**Participants**	Inclusion criteria:<br>• an elevated prostate-specific antigen(PSA) level (12.5 ng/ml)<br>• and/or abnormal digital rectal examina-tion and abnormal findings in the first prostate biopsy pathology that necessitated a repeat biopsy such as the presence of atypicalglands<br>Exclusion criteria:<br>• Saturation biopsy (24 cores) and having an indwellingurethral catheter<br>• undergone urologic surgery in the last month<br>Screened: 620 ; Eligible: 620<br>Age (mean in years ±SD): FM 61.5 (SD: 6.63); CIP 63.88 (SD: 7.27); Levofloxacin 63.23 (SD : 7.78)<br>PSA (ng/mL): not available<br>Prostate volume (cc±SD): FM 46.09 (SD: 22.57); CIP 48.98 (SD: 24.18); Levofloxacin 46.70 (SD : 25.30)<br>DM: FM 16; CIP 46; Levofloxacin 7<br>Prostate cancer: not available<br>BPH: not available
**Interventions**	Intervention (n = 104): FM;<br>• single dose of fosfomycin (orally, 3 g) the night before the procedure<br>Control (n = 526): CIP or Levofloxacin;<br>• 500 mg oral ciprofloxacin twice daily for 5 days, starting 1 day before the procedure<br>• single dose of oral 500 mg levofloxacin 60 min before the procedure
**Outcomes**	Primary outcomes:<br>Afebrile UTI<br>• fever < 38°C and dysuria accompanied by pyuria (> 10 white blood cells in 1 mm 3 of mid-stream urine)<br>Febrile UTI<br>• fever ≥ 38 ° C accompanied by one symptom of the lower urinary tract (i.e., urgency, frequency, dysuria, or suprapubic tenderness), with or without a positive urine culture<br>Secondary outcomes: not available
**Funding sources**	Not available
**Declarations of interest**	None
**Notes**	Language of publication : English

Yang 2019

**Methods**	Study design: Retrospective cohort study<br>Study dates: Not reported<br>Setting: Single center

	Country: England<br>Study endpoint: 1 month
**Participants**	Inclusion criteria:<br>• men undergoing TRUS prostate biopsy<br>Exclusion criteria:<br>• Not available<br>Screened: Not available; Eligible: 171<br>Age (mean in years ±SD): not available<br>PSA (ng/mL ±SD): not available<br>Prostate volume (cc ±SD): not available<br>DM: not available<br>Prostate cancer: not available<br>BPH: not available
**Interventions**	Intervention (n = 89): FM;<br>• one-dose Fosfomycin<br>Control (n = 82): CIP+met+genta;<br>• current combination 3-day CIP, Metronidazole and Gentamicin
**Outcomes**	Primary outcomes: infection requiring further antibiotic treatment<br>Secondary outcomes: not available
**Funding sources**	Not available
**Declarations of interest**	Not available
**Notes**	Language of publication : English

TRUS: transrectal ultrasonography; FQ: fluoroquinilone; FM: fosfomycin; PSA: prostate specific antigen; SD: standard deviation; BPH: benign prostate hyperplasia; SIRS: Systemic inflammatory response syndrome; DM: diabetes mellitus; CIP: ciprofloxacin; UTI: urinary tract infection

Characteristics of excluded studies

Hadjipavlou 2020

Reason for exclusion	Wrong intervention

Fahmy 2016

Reason for exclusion	Wrong intervention

Knaapila 2019

Reason for exclusion	Wrong intervention

Cimino 2020

Reason for exclusion	Duplicates

EUCTR 2017

Reason for exclusion	Duplicates

EUCTR 2012

Reason for exclusion	Duplicates

Refrences of excluded studies

1. Hadjipavlou M, Eragat M, Kenny C, Pantelidou M, Mulhem W, Wood C, Dall'Antonia M, Hammadeh MY. Effect of augmented antimicrobial prophylaxis and rectal swab culture–guided targeted prophylaxis on the risk of sepsis following transrectal prostate biopsy. European Urology Focus 2020;6(1):95-101.

2. Fahmy A, Rhashad H, Mohi M, Elabbadie A, Kotb A. Optimizing prophylactic antibiotic regimen in patients admitted for transrectal ultrasound-guided prostate biopsies: A prospective randomized study. Prostate International 2016;4(3):113-117.

3. Knaapila J, Gunell M, Syvänen K, Ettala O, Kähkönen E, Lamminen T, Seppänen M, Jambor I, Rannikko A, Riikonen J, Munukka E, Eerola E, Hakanen AJ, Boström PJ. Prevalence of complications leading to a health care contact after transrectal prostate biopsies: A prospective, controlled, multicenter study based on a selected study cohort. European Urology Focus 2019;5(3):443-448.

4. Cimino S, Verze P, Venturino L, Alessio P, Migliara A, Imbimbo C, Mirone V, Russo GI, Morgia G. Complication rate after antibiotic prophylaxis with fosfomycin versus fluorochinolones or β-lactam antibiotics in patients undergoing prostate biopsy: A propensity score-adjusted analysis. European Urology Focus 2020;6(2):370-375.

5. Fosfomycin vs ciprofloxacin for transrectal biopsy - a randomized trial.
https://trialsearch.who.int/Trial2.aspx?TrialID=EUCTR2017-000772-28-SE

6. Randomized controlled trial of the efficacy of fosfomycin vs ciprofloxacin as antibiotic prophylaxis before transrectal ultrasound guided prostate.
https://trialsearch.who.int/Trial2.aspx?TrialID=EUCTR2012-001031-31-ES

Characteristics of ongoing studies

EUCTR2012-001031-31-ES 2012

**Study name**	Randomized controlled trial of the efficacy of fosfomycin vs ciprofloxacin as antibiotic prophylaxis before transrectal ultrasound guided prostate
**Methods**	Study design: randomised parallel controlled trial (open label)<br>Study dates: not reported<br>Setting: outpatients<br>Country: Spain
**Participants**	Inclusion criteria:<br>• Age over 18 years<br>• A patient who attends hospital outpatient prostate biopsy by PSA over 4 ng / ml or suspicious tumor<br>• Subjects who have given written informed consent to participate in the study<br>Exclusion criteria:<br>• History (confirmed or suspected) allergy to any of the drugs included in the study<br>• History (confirmed or suspected) of intolerance to any of the drugs included in the study<br>• Presence of urinary tract infection confirmed by urine culture or urinalysis<br>• Clinical findings suggestive of infection (any origin)<br>• Antibiotic therapy in the last 4 weeks.<br>• Patient carrier catheter<br>Estimated enrollment: 470
**Interventions**	Intervention: single dose of fosfomycin 3 g one hour before the biopsy<br>Control: ciprofloxacin 500 mg 1 hour before the biopsy
**Outcomes**	Primary outcomes:<br>• Bacteriuria Appearance in the urine culture of a significant number of bacteria (> 10^5 cfu / ml).<br>Secondary outcomes:<br>• Urinary tract infection<br>• Genitourinary infection associated with fever (> 38 ° C)<br>• Sepsis<br>• Pathogens present in urine and their sensitivity to antibiotics<br>• Bacteremia<br>• Clinical and demographic variables
**Starting date**	04/06/2012
**Contact information**	Javier Júdez Gutiérrez; javier.judez@ffis.es
**Notes**	Sponsor: Fundación para Formación e Investigación Sanitaria<br>Source: https://trialsearch.who.int/Trial2.aspx?TrialID=EUCTR2012-001031-31-ES

EUCTR2017-000772-28-SE 2017

**Study name**	Fosfomycin vs ciprofloxacin for transrectal biopsy - a randomized trial
**Methods**	Study design: randomised parallel controlled trial (open label)<br>Study dates: september 2015 to December 2020<br>Setting: not reported<br>Country: Sweden
**Participants**	Inclusion criteria:<br>• Indication for prostate biopsy<br>Exclusion criteria:

	• Allergy to Fosfomycin or Ciprofloxacin<br>• Use of Tizanidine<br>• Hemodialysis or severe renal failure<br>Estimated enrollment: 3448
**Interventions**	Intervention: fosfomycin 3 g<br>Control: ciprofloxacin 500 mg
**Outcomes**	Primary outcomes:<br>• Sepsis<br>Secondary outcomes:<br>• Prescribed antibiotics for UTI within 30 Days after the biopsy
**Starting date**	16/05/2017
**Contact information**	Johan Styrke; johan.styrke@umu.se
**Notes**	Sponsor: Västernorrland county coucil<br>Source: https://www.clinicaltrialsregister.eu/ctr-search/trial/2017-000772-28/SE

NCT03228108

**Study name**	Culture-guided Antimicrobial Prophylaxis in Men Undergoing Prostate Biopsy (pro-SWAP)
**Methods**	Study design: randomised parallel controlled trial (open label)<br>Study dates: 03/04/2018 – 26/09/2021<br>Setting: outpatients<br>Country: Netherlands
**Participants**	Inclusion criteria:<br>• Subject is able and willing to sign the Informed Consent Form<br>• A Subject undergoes a transrectal prostate biopsy as part of the standard care in the Radboudumc (Nijmegen), Canisius Wilhelmina hospital (Nijmegen) or Catharina hospital (Nijmegen) (because of suspicion of prostate cancer)<br>• 18 Years to 90 Years (Adult, Older Adult)<br>Exclusion criteria:<br>• Inability to receive ciprofloxacin (e.g. documented history of sensitivity to medicinal products or excipients similar to those found in the antibiotic prophylaxis, relevant history or presence of cardiovascular disorders)<br>• Inability to receive either co-trimoxazole, fosfomycin and pivmecillinam/augmentin prophylaxis for any reason (e.g. documented history of sensitivity to medicinal products or excipients similar to those found in the antibiotic prophylaxis)<br>• Inability to understand the nature of the trial and the procedures required<br>• Individuals with an urinary tract infection or acute prostatitis within 14 days prior to intervention<br>• Individuals who receive antibiotics within 14 days before prostate biopsy<br>• Individuals who fail to send a rectum swab to the microbiology laboratory<br>• Individuals whose rectal swab shows no growth on a (growth) control MacConkey agar without antibiotics<br>Estimated enrollment: 1400
**Interventions**	Intervention:

	• trimethoprim/sulfamethoxazole (SXT) 960 mg orally 2 hours before and 12 hours after prostate biopsy or<br>• fosfomycin 3 g 3 hour before the biopsy or<br>• pivmecillinam/augmentin respectively 400 mg and 500/125 mg 2 hours before prostate biopsy followed by 2 days with three divided doses each day after prostate biopsy<br>Control:<br>• ciprofloxacin 500 mg orally 2 hours before the biopsy and 12 hours after transrectal prostate biopsy
**Outcomes**	Primary outcomes:<br>• Any registered clinical infectious complication after prostate biopsy [Time Frame: within 7 days post-biopsy]; Urinary tract infection, pyelonephritis, sepsis, fever, acute prostatitis, acute epididymitis<br>Secondary outcomes:<br>• Cost of care [Time Frame: within 30 days after prostate biopsy] Difference of costs between the intervention and the control group<br>• Positive microbiological results [Time Frame: within 7 and 30 days after prostate biopsy]<br>Urine or blood culture results<br>• Any registered clinical infectious complication after prostate biopsy [Time Frame: within 7 and 30 days after prostate biopsy] Urinary tract infection, pyelonephritis, sepsis, fever, acute prostatitis, acute epididymitis<br>• Hospitalization after prostate biopsy [Time Frame: within 30 days after prostate biopsy]<br>Any hospital admission, including ICU admission<br>• Overall mortality [Time Frame: within 30 days after prostate biopsy]<br>Mortality of any cause<br>• Side effects of used antibiotics [Time Frame: within 30 days after prostate biopsy]<br>All side effects mentioned in the Summary of Product Characteristics (SPC)<br>• Prevalence of ciprofloxacin-resistant gram negative bacilli in local rectal flora [Time Frame: rectal swabs are taken 14 days before biopsy]<br>Assessed through microbiological rectal swab cultures<br>• Overall antibiotic use after prostate biopsy [Time Frame: within 30 days after prostate biopsy]<br>Number of antibiotic prescriptions
**Starting date**	03/04/2018
**Contact information**	Heiman Wertheim; Radboud University Medical Center
**Notes**	Sponsor: not reported<br>Source: https://ClinicalTrials.gov/show/NCT01803191

RBR-9tk9pwz

**Study name**	Study comparing the efficacy of fosfomycin and ciprofloxacin antibiotics in the antibiotic prophylaxis of transrectal prostate biopsy
**Methods**	Study design: randomised parallel controlled trial (open label)<br>Study dates: not reported<br>Setting: outpatients

	Country: Brazil
**Participants**	Inclusion criteria:<br>• male gender<br>• have a formal indication for prostate biopsy<br>• aged between 18 and 100 years.<br>Exclusion criteria:<br>• patients who for some reason cannot take the proposed medications<br>• patients with a history of severe bleeding complications during the biopsy procedure<br>Estimated enrollment: 115
**Interventions**	Intervention:<br>• fosfomycin trometamol in a single dose of 3 grams, orally, three hours before the biopsy<br>Control:<br>• ciprofloxacin 500 mg 2 hours before the biopsy
**Outcomes**	Primary outcomes:<br>• Occurrence of infectious complications, which include genitourinary infection (symptoms of fever, chills and dysuria) with or without fever and asymptomatic bacteriuria, evaluated after the patients' return visit, in the medical record, and urine culture performed 10 days after the biopsy procedure, in each group, through the relative and absolute frequency of the occurrence<br>Secondary outcomes:<br>• Prostate Specific Antigen (PSA) level<br>• Previous use of a urinary catheter<br>• Uroculture and bacterial resistance 10 days after biopsy in patients who had symptoms of infectious complications<br>• The use of antibiotics in the last three months<br>• The personal history of Diabetes Melitus<br>• The result of the anatomopathological study of the participants' prostate biopsies
**Starting date**	08/04/2018
**Contact information**	Eduardo Gomes; eduardoverrigomes@outlook.com
**Notes**	Sponsor: Universidade Federal do Paraná - Campus Toledo<br>Source: https://ensaiosclinicos.gov.br/rg/RBR-9tk9pwz

Search strategies

Ovid MEDLINE(R) and Epub Ahead of Print, In-Process & Other Non-Indexed Citations via Ovid (Daily 1946 to 14 January 2022)

1 exp Prostate/pa [Pathology]
2 exp Prostatic Diseases/pa [Pathology]
3 (Prostat* and biops*).mp.
4 1 or 2 or 3
5 exp Fosfomycin/
6 (fosfomycin* or fosfocil or fosfocin* or fosfocina or fosfocine or fosfomicin* or fosfonomycin* or fosmycin* or infectofos or "mk 0955" or mk 955 or mk0955 or mk955 or phosphomycin* or phosphonomycin*).mp.
7 23155-02-4.mp.
8 5 or 6 or 7
9 4 and 8

Embase Classic+Embase via Ovid (1947 to 14 January 2022)

#1 'prostate'/exp AND 'pathology'/exp
#2 'prostate disease'/exp AND 'pathology'/exp
#3 prostat*:ti,ab AND biops*:ti,ab
#4 #1 OR #2 OR #3
#5 'fosfomycin'/exp
#6 fosfomycin*:ti,ab OR fosfocil:ti,ab OR fosfocin*:ti,ab OR fosfocina:ti,ab OR fosfocine:ti,ab OR fosfomicin*:ti,ab OR fosfonomycin*:ti,ab OR fosmycin*:ti,ab OR infectofos:ti,ab OR 'mk 0955':ti,ab OR 'mk 955':ti,ab OR mk0955:ti,ab OR mk955:ti,ab OR phosphomycin*:ti,ab OR phosphonomycin*:ti,ab
#7 '23155 02 4':rn
#8 #5 OR #6 OR #7
#9 #4 AND #8

Cochrane Library via Wiley

#1 [mh "Prostate"/PA]
#2 [mh "Prostatic Diseases"/PA]
#3 #1 or #2 or #3
#4 [mh "Fosfomycin"]
#5 (fosfomycin* or fosfocil or fosfocin* or fosfocina or fosfocine or fosfomicin* or fosfonomycin* or fosmycin* or infectofos or "mk 0955" or mk 955 or mk0955 or mk955 or phosphomycin* or phosphonomycin*):ti,ab,kw
#6 23155-02-4:ti,ab,kw
#7 #5 or #6 or #7
#8 #5 or #6 or #7
#9 #4 and #8

Web of Science

#1 TS=(prostat* AND biops* AND (fosfomycin* OR fosfocil OR fosfocin* OR fosfocina OR fosfocine OR fosfomicin* OR fosfonomycin* OR fosmycin* OR infectofos OR "mk 0955" OR "mk 955" OR mk0955 OR mk955 OR phosphomycin* OR phosphonomycin*))

ClinicalTrials.gov

Condition: prostate
Intervention: fosfomycin

WHO ICTRP

1. prostate AND fosfomycin

KOREAMED

1. (prostate[TIAB] OR prostates[TIAB] OR prostatic[TIAB]) AND (fosfomycin[TIAB] OR fosfocil[TIAB] OR fosfocin[TIAB] OR fosfocina[TIAB] OR fosfocine[TIAB] OR fosfomicin[TIAB] OR fosfonomycin[TIAB] OR

fosmycin[TIAB] OR infectofos[TIAB] OR "mk 0955"[TIAB] OR "mk 955"[TIAB] OR mk0955[TIAB] OR mk955[TIAB] OR phosphomycin[TIAB] OR phosphonomycin[TIAB])

KMBASE

1 prostat* OR 전립선
2 fosfomycin*
3 1 AND 2
